# Inhibition of the inferior parietal lobe triggers state-dependent network adaptations

**DOI:** 10.1016/j.heliyon.2024.e39735

**Published:** 2024-10-23

**Authors:** Kathleen A. Williams, Ole Numssen, Juan David Guerra, Jakub Kopal, Danilo Bzdok, Gesa Hartwigsen

**Affiliations:** a Lise Meitner Research Group Cognition and Plasticity, Max Planck Institute for Human Cognitive and Brain Sciences, Leipzig, Germany; b Methods and Development Group “Brain Networks”, Max Planck Institute for Human Cognitive and Brain Sciences, Leipzig, Germany; c The Neuro - Montreal Neurological Institute (MNI), McConnell Brain Imaging Centre, Department of Biomedical Engineering, Faculty of Medicine, School of Computer Science, McGill University, Montreal, Canada; d Mila - Quebec Artificial Intelligence Institute, Montreal, Quebec, Canada; e Wilhelm Wundt Institute for Psychology, Leipzig University, Germany

**Keywords:** TMS, cTBS, fMRI, Functional connectivity, Cognition, Resting state

## Abstract

The human brain comprises large-scale networks that flexibly interact to support diverse cognitive functions and adapt to variability in daily life. The inferior parietal lobe (IPL) is a hub of multiple brain networks that sustain various cognitive domains. It remains unclear how networks respond to acute regional perturbations to maintain normal function. To provoke network-level adaptive responses to local inhibition, we combined offline transcranial magnetic stimulation (TMS) over left or right IPL with neuroimaging during attention, semantic and social cognition tasks, and rest. Across tasks, TMS specifically affected task-active network activity with inhibition and facilitation. Network interaction responses differed between rest and tasks. After TMS over both IPL regions, large-scale network interactions were exclusively facilitated at rest, but mainly inhibited during tasks. Overall, responses to TMS primarily occurred in and between domain-general default mode and frontoparietal subnetworks. These findings elucidate short-term adaptive plasticity in response to network node inhibition.

## 1 Introduction

Efficient human cognition relies on the dynamic interaction of large-scale networks comprising spatially distributed cortical and subcortical regions [1,2]. One particularly unique network is the default mode network (DMN), a set of functionally connected regions that systematically deactivate during externally oriented tasks, but activate during resting state and self-referential thinking [[3], [4], [5]]. The inferior parietal lobe (IPL), part of the evolutionarily expanding association cortex, is central to multiple human functional networks [[6], [7], [8]], including the DMN. As a multimodal convergence zone, the IPL critically contributes to brain function across cognitive domains, including attention [9,10], language [11,12], decision making [13], and social cognition [11,14]. While the IPL often contributes bilaterally, hemispheric functional asymmetries are also present [[15], [16], [17]]. Most notably, left IPL is a part of the highly-lateralized language network [12], while visuospatial attention receives greater contributions from the right IPL [18]. The importance of the IPL for daily functioning, and the DMN in particular, is reinforced by neuropsychological dysfunction rooted, at least partially, in aberrant functional connectivity [19]. However, probing the mechanisms by which such networks flexibly adapt is a non-trivial methodological challenge.

Transcranial magnetic stimulation (TMS) is a useful non-invasive tool to probe network interactions, as it can trigger short-term reallocation of resources by temporarily perturbing neuronal activity. This allows the investigation of causal brain-behavior relationships [20,21]. TMS effects are well-known to extend beyond the targeted area to structurally and functionally connected regions [22,23]. Combining TMS with functional neuroimaging is a promising approach to understanding how networks functionally adapt [24,25]. For example, applying low- (1 Hz) or high-frequency (20 Hz) repetitive TMS over the IPL DMN hub was shown to increase and decrease resting-state connectivity to distant network nodes respectively [26]. Further, continuous theta-burst stimulation (cTBS) is thought to inhibit neural activity in the motor cortex and related networks for at least 30 min after stimulation [27]. Similar effects also seem to apply to other cortical networks, however supporting evidence is variable [[28], [29], [30]]. Remote network effects have been found when stimulating central language or memory network nodes and linked to changes in task performance [31,32]. In the language study, the targeted network itself was inhibited while potential compensatory upregulation was observed in neighboring areas [31] (see Hartwigsen et al. [33]). Because TMS studies of brain networks have been conducted primarily during rest, it is unclear if this pattern of local inhibition and compensatory upregulation also applies during cognitive tasks. Moreover, task-related network effects have rarely been explored across different cognitive domains.

**Fig. 1:**
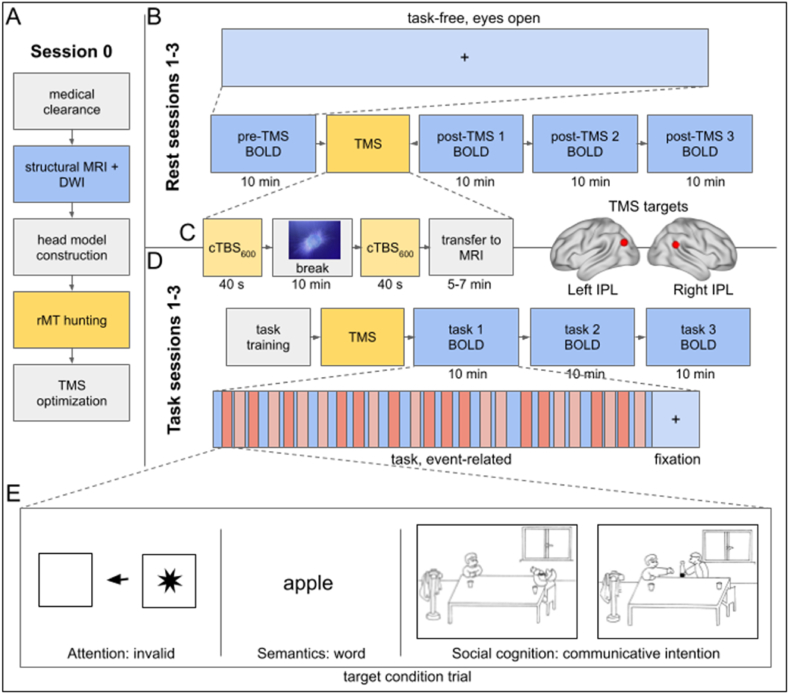
Experimental Design. The experiment consisted of six experimental sessions for every participant with an optional initial session (A) for acquiring structural and diffusion-weighted images (DWI) or TMS motor thresholds. (B) Resting-state measurements comprised one pre-stimulation and three post-stimulation fMRI runs in which subjects visually focused on a fixation cross in the center of their field of view for 10 min. (C) Left, right, or sham TMS was administered to the IPL (figure right) as two inhibitory continuous theta-burst stimulation (cTBS) pulse trains at 90 % resting motor threshold (rMT) spaced apart by 10 min, during which participants silently watched the Inscapes video (left). (D) Task sessions included three post-stimulation fMRI runs in which three tasks were presented separately in a pseudo-randomized order. All tasks presented a target condition that recruits attentional reorienting, semantic processing, or social cognition and one well-matched control condition as a contrast. Stimuli were presented in an event-related fashion and included rest conditions at the end for a complete 10-min run. (E) Example target condition stimuli are displayed for attention invalid (left), semantics word (center), and social cognition communicative intention (right). For rest and for task, left IPL TMS, right IPL TMS, and sham TMS were delivered in three sessions scheduled in a pseudo-randomized order at least one week apart. BOLD, blood-oxygen-level-dependent fMRI.

**Fig. 2:**
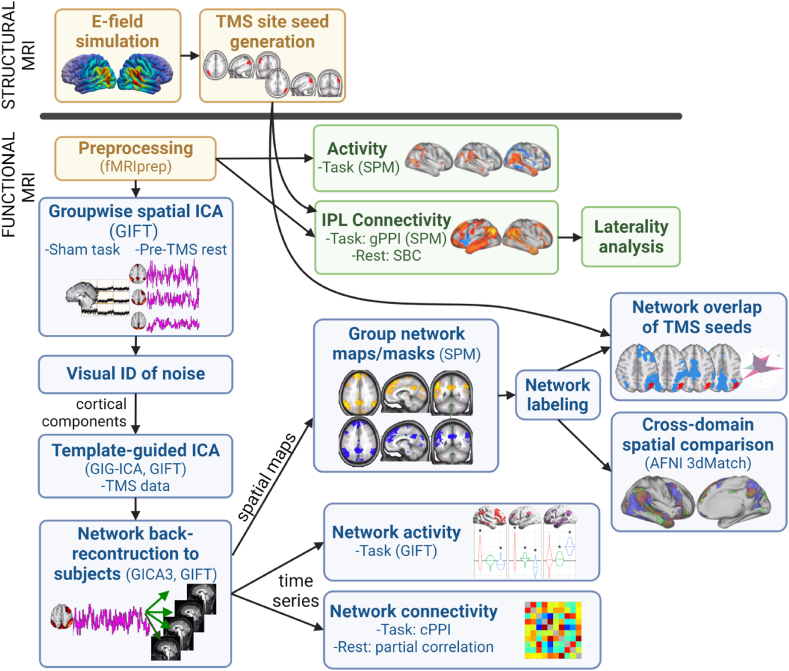
Analysis overview. Prior to fMRI measurements, electric fields (E-fields) were simulated for each subject to plan TMS targets. The field maps were used to generate subject-specific TMS site seeds used for analysis. Functional data analysis consisted of voxelwise and network-level analysis pipelines. Voxelwise analyses (green panels) included univariate activation and whole-brain seed-based connectivity (SBC), which was further assessed for hemispheric preferences using laterality estimations. Large-scale networks were derived using independent component analysis (ICA) for further spatial and temporal characterization (blue panels). Network analyses were carried out in the spatial domain to assess the relationship of TMS targets to network topography across cognitive domains. Network time series were analyzed for TMS effects on task-specific activity and between-network connectivity.

To fill this gap, the present study explored how functional brain networks adapt to short-term perturbation (via TMS) of a key cognitive hub, while maintaining normal behavior. Specifically, using tasks representing three cognitive domains, we tested whether stimulation-induced network modulations are specific to hemispheric functional specialization. Following our previous fMRI study on hemisphere-specific functional organization of the IPL [17], we investigated domain-specific differences in the adaptive functional interactions between large-scale networks after cTBS to the left or right IPL. Combining offline, spaced double cTBS with fMRI measurements in task-active attention, semantics, and social cognition or task-free resting state (experimental design, Fig. 1), we characterized the functional connectivity of the IPL and large-scale networks and their responses to IPL inhibition in each domain (analysis overview, Fig. 2).

If normal behavior persisted after network perturbation by TMS, and a unique response was observed for the left and right hemispheres for a given task, it would suggest the changes are in fact adaptive and influenced by underlying hemispheric specialization. Explicitly, based on our previous study [17], we hypothesized that TMS over the right IPL would suppress activity in task-relevant networks, such as dorsal and ventral attention networks, during attentional reorientation. We expected that TMS over the left IPL would parallel these effects in semantic networks during lexical decision making. For social cognition, we expected similar responses to left and right IPL stimulation, such that inhibiting a node of task-active networks would trigger upregulation in homologous regions or in adjacent interacting lower-order networks. We anticipated left and right IPL inhibition to evoke similar alterations localized to DMN during unconstrained cognitive activity in the resting state.

## 2 Results

As expected, no behavioral modulations from TMS were detected (Fig. S1, Table S1).

In the functional data analyses, first we present whole-brain voxelwise results, depicted in the green panels in Fig. 2. We report baseline activity and TMS effects across tasks as well as baseline and TMS-modulated connectivity with IPL targets during resting state. We computed baseline functional connectivity with left IPL and right IPL in each task and used the voxelwise maps for laterality calculations to compare and report hemisphere-specific whole-brain IPL connectivity features across domains without TMS.

Following voxelwise results, we report spatial and temporal results for large-scale networks derived using independent component analysis (ICA) (blue panels, Fig. 2). Spatially, we overlapped baseline group-level network masks across domains and across TMS targets to compare domain-specific network topographies. ICA time series were used to estimate network activity during the tasks to identify task-specific baseline activity and TMS effects. Finally, we describe TMS modulations of between-network connectivity across cognitive domains.

### 2.1 TMS modulation of task-related activity is generally similar across target hemispheres

**Fig. 3:**
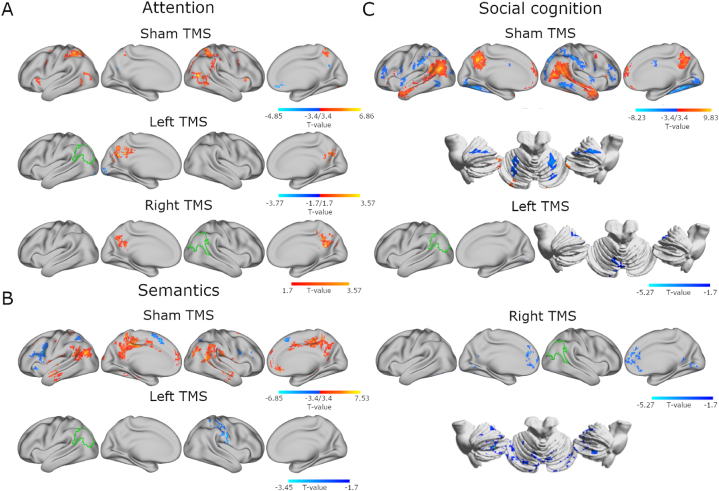
TMS-induced activity modulation is more similar across hemispheres than across cognitive domains. (A) Results for the attention task, contrasting invalid with valid conditions after sham TMS for baseline activity (top row), and for TMS effects, estimated by contrasting left (middle row) or right (bottom row) TMS with sham in the target invalid condition. (B) Results for activity in semantics, contrasting words with pseudowords after sham TMS for baseline activity (top row), and for TMS effects in the target word condition, contrasting left TMS (bottom row) with sham. No right TMS effects were detected for semantic activity in univariate analyses. (C) Results for baseline activity during social cognition, from contrasting communicative intention with physical causality conditions after sham TMS (top row), and for TMS effects in the target communicative intention condition, contrasting left (middle row) or right (bottom row) TMS with sham. Stimulated hemisphere is indicated with green outlines of the group-level representation seeds. Baseline results are FWE-corrected at cluster level p < 0.05 after voxel thresholding at uncorrected p < 0.001 (t > 3.42). TMS results are FWE-corrected at cluster level p < 0.05 after voxel thresholding at uncorrected p < 0.05 (t > 1.7).

We first assessed baseline activation patterns using voxelwise general linear model (GLM) univariate analyses. Across cognitive domains without active TMS, target versus control contrasts identified baseline whole-brain activation (Fig. 3, SI Table S2 for details). Attentional reorientation showed bilateral activation with stronger and more spatially extended contributions in the right hemisphere compared to the left hemisphere (Fig. 3a). Recruited regions comprised parts of dorsal and ventral attention networks, including bilateral anterior insula and temporo-parietal areas such as superior parietal lobule (SPL) and posterior temporal gyri. Deactivation occurred in medial nodes of the DMN, anterior (ACC) and posterior cingulate cortices (PCC). The lexical decision task activated a recognizable semantic network that includes bilateral IPL, temporal gyri, ACC, and precuneus. Left frontal cortex, especially the inferior frontal gyrus, showed notable contrast-specific deactivation (Fig. 3b). Social cognition had the strongest effect sizes among the tasks, activating a classical theory of mind network that, like the semantic network, consists of many DMN regions, generally precuneus and extending through bilateral temporo-parietal cortices (Fig. 3c). Bilateral regions of the cerebellum were also recruited. Deactivations occurred bilaterally in the middle frontal gyri and throughout many dorsal attention and visual network areas.

Next, we tested for TMS effects on task-specific activity using the univariate maps. To locate TMS-induced modulation of neural activity in each domain, we compared target responses in active (left or right) and sham TMS conditions for each task. Considering the TMS effects on activity overall, we found that changes in activation varied across tasks more than across hemispheres of perturbation (Fig. 3, SI Table S3). In general, we did not find strong stimulation effects in the targeted areas. For attention, both left and right TMS increased activity in a medial cluster across precuneus and PCC (Fig. 3a). Left-hemisphere TMS also inhibited activity in the occipital pole and left cerebellum. For semantics, only left TMS decreased activation in a large right-hemisphere cluster encompassing parts of supramarginal gyrus (SMG), precentral gyrus, superior temporal gyrus, IPL, and SPL (Fig. 3b). In social cognition, left and right TMS inhibited target condition responses, mostly in bilateral cerebellum and lingual gyri (Fig. 3c). Right-hemisphere TMS induced more extensive inhibition throughout the cerebellum and additionally reduced activation in ACC and PCC.

### 2.2 TMS reduces connectivity between the IPL and distributed areas at rest

**Fig. 4:**
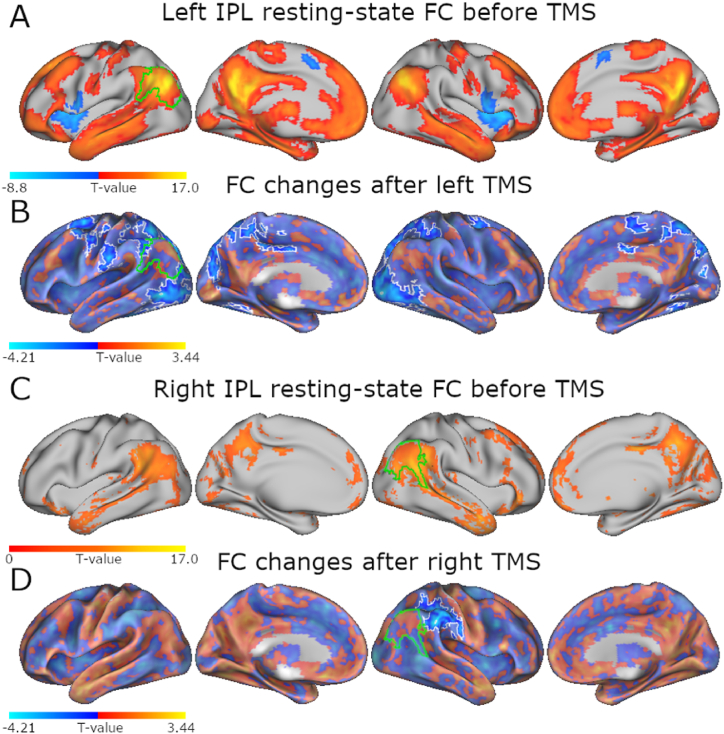
Resting-state connectivity with TMS targets decreased after stimulation. Resting-state functional connectivity at baseline (before TMS) for (A) left IPL and (C) right IPL show the TMS targets as connected to the larger DMN at the group level. Green outlines indicate group-level representations of the TMS sites seeded for connectivity. (B) and (D) show TMS-induced changes in connectivity to left IPL after left TMS and to right IPL after right TMS, respectively. TMS induced distributed disruptions in regional connectivity, specifically showing decreased connectivity from the IPL to beyond-target regions that were already anticorrelated with the stimulated area at baseline. We determined TMS effects by contrasting the average difference of pre- and post-stimulation connectivity maps for active TMS against sham, matching the hemisphere IPL seeds to the active TMS conditions. Baseline results are FWE-corrected at cluster level p < 0.05 after voxel thresholding at uncorrected p < 0.001 (t > 3.4). TMS results are FWE-corrected at cluster level p < 0.05 after voxel thresholding at uncorrected p < 0.05 (t > 1.7).

To estimate TMS-induced changes in intrinsic functional connectivity (FC) between the stimulation targets and the rest of the brain, we used resting-state seed-based connectivity and compared pre-post TMS connectivity differences in active and sham conditions. At baseline, left IPL and right IPL connect with the greater DMN. Specifically, before TMS, left and right IPL both correlated with DMN regions including major anterior and posterior midline structures and bilateral temporo-parietal cortices. In contrast to right IPL connectivity, left IPL showed distinctive anticorrelations in bilateral insula, inferior frontal gyri, and supplementary motor areas (Fig. 4).

Overall, TMS reduced intrinsic connectivity to IPL primarily in regions with weak or negative connections to IPL at baseline (See SI Table S4 for details). Both left and right TMS induced decreased within-IPL connectivity in the stimulated hemisphere. Additionally, after left-hemisphere TMS, IPL connectivity decreased bilaterally throughout regions of the dorsal attention network including the cerebellum. Right-hemisphere TMS inhibited IPL connectivity to within-hemisphere regions anterior to the target, namely anterior IPL, SMG and precentral gyrus. These IPL connectivity results indicate that regionally-specific FC modulations depend on baseline connectivity and underlying networks.

### 2.3 Baseline balance of whole-brain functional connectivity with IPL in each hemisphere changes with cognitive domain

**Fig. 5:**
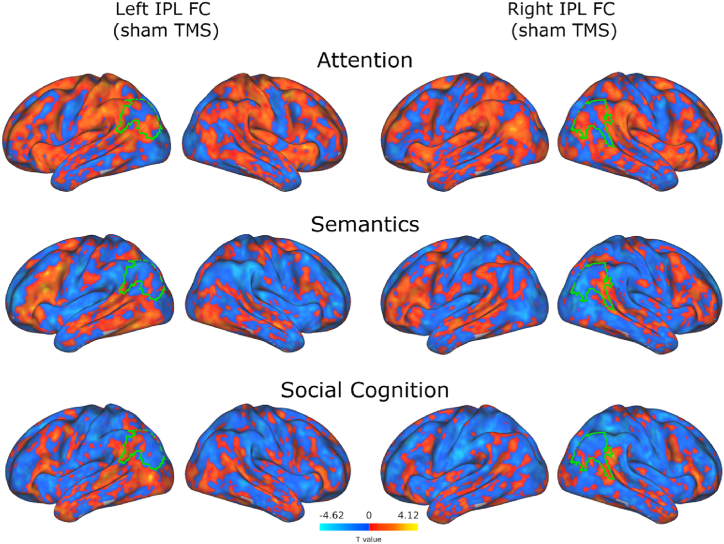
Functional connectivity to IPL regions is hemisphere-specific and varies across cognitive domain. To illustrate the sources of the laterality analyses, group-level task-specific IPL connectivity (gPPI) maps are displayed for each TMS site seed (left IPL in left columns, right IPL in right columns) across the tasks (one-sided t-tests). Laterality measures were computed at the subject level using voxelwise left and right IPL FC maps from the baseline condition. Green outlines of group-level representations indicate seeds.

**Fig. 6:**
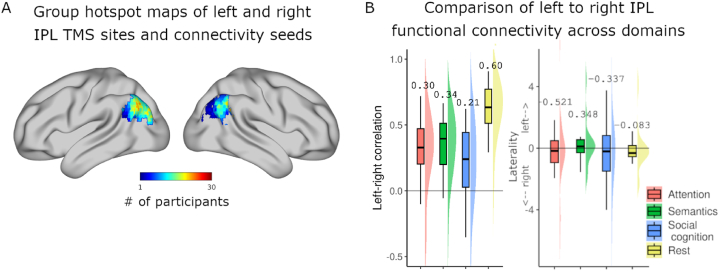
Relating domain-specific connectivity to IPL TMS targets. (A) Electric field hotspots across participants for left and right TMS generated by summing subject-level TMS site seeds for each hemisphere. Values display voxelwise overlap across the group for each seed. (B) Comparison of left versus right IPL-to-whole-brain functional connectivity (FC) across domains in baseline conditions. For each cognitive domain, sham or pre-stimulation left IPL and right IPL FC maps were correlated, within subject, to estimate their similarity in each state (left plot). Values displayed in plots are the group means of correlations and laterality indices (right plot) in each domain. Resting-state FC is the most similar between hemispheres, while social cognition is the most differentiated. Laterality indices show attention and social cognition to have stronger right IPL connectivity, semantics to have greater left IPL connectivity, and rest to be relatively balanced.

To further explore the underlying connectivity influencing domain-specific TMS modulations, we investigated the relationship between left and right IPL connectivity within each domain at baseline (Fig. 6B). To this end, we estimated left and right IPL FC using voxelwise seed-based connectivity analysis of pre-TMS rest data (Fig. 4) and generalized psychophysiological interactions (gPPI) analysis of sham task data (Fig. 5). We directly compared left IPL connectivity to right IPL connectivity within each domain by correlating left and right connectivity maps and calculating whole-brain laterality indices (details in Methods). Spatial correlation of IPL connectivity maps estimates the similarity of each TMS target's connectivity to the rest of the brain and laterality indices indicate whether left or right IPL has greater connectivity to the rest of the brain. Hemispheric IPL connectivity indices across cognitive domains showed patterns related to typical activations evoked in each domain. Attention and social cognition, known to recruit more right-hemisphere activity, had connectivity preferences to right IPL, exhibited by negative laterality indices of −0.34 and −0.52, respectively. Likewise, semantic cognition primarily engages a left-lateralized network, and while left-right IPL FC was relatively similar (r = 0.34), the average laterality index (LI = 0.35) indicates stronger whole-brain FC with left IPL than with right IPL. Among cognitive domains, left and right IPL connectivity were most similar during rest, with the highest correlation (r = 0.60) accompanied by a near-zero laterality index (LI = −0.08). With the lowest left-right connectivity correlation (r = 0.21), social cognition showed the least symmetric IPL connectivity.

### 2.4 Networks organize differently across domains at baseline

**Fig. 7:**
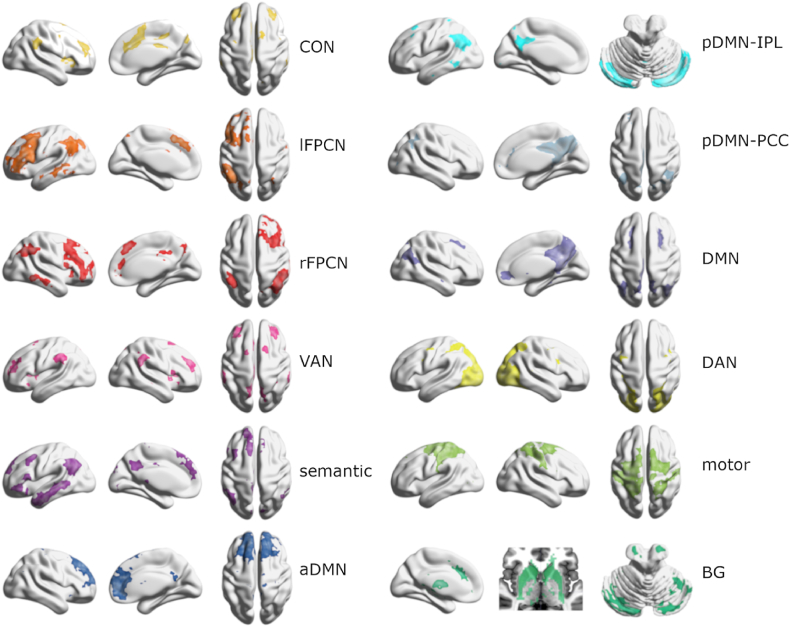
Large-scale networks of interest investigated in our study. (A) Maps of the selected connectivity networks derived from one-sided t-tests of pre-stimulation rest data (uncorrected voxel-level threshold p < 0.001 and FWE-corrected cluster level p < 0.05), see Fig. S2 for domain-specific masks overlaid for each network. CON, cingulo-opercular network; lFPCN, left frontoparietal control network; rFPCN, right frontoparietal control network; VAN, ventral attention network; semantic network; aDMN, anterior default mode network; pDMN-IPL, posterior default mode IPL subnetwork; pDMN-PCC, posterior default mode posterior cingulate cortex subnetwork; DMN, default mode network; DAN, dorsal attention network; motor network; BG, basal ganglia network.

To identify large-scale brain networks that emerge across the cognitive domains, we defined networks in rest, attention, semantics, and social cognition separately using groupwise spatial ICA of pre-TMS or sham data. Our selected networks of interest included higher order, basal ganglia, and motor networks (Fig. 7). ICA networks that were resolved within each domain were well-matched across rest and task states, however not all networks were resolved across all domains. Resting-state ICA generated the most (12) networks of interest, including the cingulo-opercular network (CON), left and right frontoparietal control networks (l/rFPCN), ventral and dorsal attention networks (VAN and DAN), semantic network, classical default mode network, and DMN subnetworks named by distinctive regions of correlation, specifically IPL (posterior (p)DMN-IPL), posterior cingulate cortex (pDMN-PCC), and anterior DMN (aDMN). Notably, pDMN-IPL included substantial bilateral cerebellum contributions. In attention, pDMN-PCC and CON did not resolve distinctly, yielding ten networks of interest. The fewest (9) networks segregated during semantics, excluding the CON and both posterior DMNs. Interestingly, for the semantic task, the spatial extent of lFPCN was substantially reduced relative to the other domains. Social cognition lacked only pDMN-PCC from the resting-state network set. We spatially matched the sets of task networks to the full set of resting-state networks using weighted spatial correlations (Table 1), demonstrating that most of the same networks persist across domains but with variable spatial topographies (see also masks of each network overlapping for all domains in Supplemental Information, Fig. S2).

**Table 1:** Network match and similarity across domains.

rest networks		attention networks		weighted corr.	semantics networks		weighted corr.	social cognition networks		weighted corr.
1	lFPCN	1	lFPCN	0.705	1	lFPCN	0.025	1	lFPCN	0.641
2	rFPCN	2	rFPCN	0.703	2	rFPCN	0.679	2	rFPCN	0.686
3	VAN	3	VAN	0.439	3	VAN	0.551	3	VAN	0.324
4	Semantic	4	Semantic	0.637	4	Semantic	0.536	4	Semantic	0.592
5	aDMN	5	aDMN	0.622	5	aDMN	0.598	5	aDMN	0.689
6	pDMN-IPL	6	pDMN-IPL	0.525	–	–	–	6	pDMN-IPL	0.523
7	pDMN-PCC	–	–	–	–	–	–	–	–	–
8	DMN	7	DMN	0.458	6	DMN	0.592	7	DMN	0.357
9	DAN	8	DAN	0.497	7	DAN	0.39	8	DAN	0.402
10	Motor	9	Motor	0.351	8	Motor	0.184	9	Motor	0.312
11	BG	10	BG	0.525	9	BG	0.655	10	BG	0.636
12	CON	–	–	–	–	–	–	11	CON	0.45

Table 1. The table displays the list of resting-state networks-of-interest that were used as a reference set to match networks across domains (Column 1), followed by the matched networks in each task and the weighted 3D correlations of the unthresholded maps to the references. See Fig. 7 caption for abbreviations.

### 2.5 Spatial relationships of TMS sites vary with domain-specific networks

**Fig. 8:**
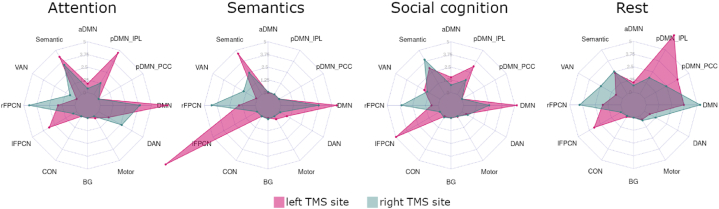
TMS site overlap varies with domain-specific networks. Spider plots show the quantity of group-level E-field hotspot overlap with each ICA network in each domain (displayed in Fig. S2), quantified by overlapping binary masks of the left and right group TMS site hotspots on each network mask and reported as percentage of the network.

To spatially relate left and right IPL TMS targets to the identified networks across domains, we quantified the overlap of the group-level TMS site hotspots (Fig. 6A) with masks of each network for all domains. Spider plots of TMS hotspot network overlap show that the networks with the greatest E-field overlap are different across domains (Fig. 8). This suggests that the spatial reconfiguration of networks during cognition changes network TMS exposure across domains. Specifically, for the left IPL TMS site, DMN had the greatest overlap during attention and social cognition, lFPCN during semantics, and pDMN-IPL during rest. For the right IPL TMS site, rFPCN had the greatest overlap during attention and semantics, the semantic network had the greatest overlap during social cognition, and DMN overlapped the most during rest.

Thus far, these results demonstrate a strong domain-specificity in the functional connectivity of the stimulated regions.

### 2.6 TMS modulates task-specific network activity

**Fig. 9:**
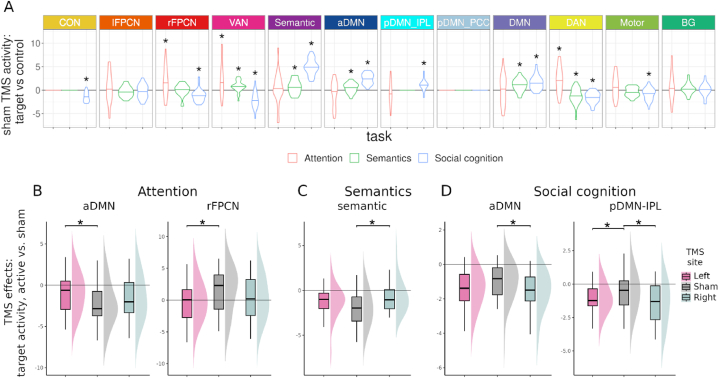
TMS modulates task-specific network activity in each domain. (A) Networks exhibit task-specific activity. For each network that was resolved within a task, the mean difference between target and control condition beta weights from the regression analysis are plotted for attention (red), semantics (green), and social cognition (blue). Significantly active networks in each task are marked with an asterisk, indicating Bonferroni-Holm-corrected p < 0.05 in pairwise permutation tests (n = 10,000). (B) Contrasting left IPL TMS versus sham during attention revealed altered network activity, reducing deactivation in aDMN and activation in rFPCN. For all tasks, performance remained unchanged. (C) Contrasting right IPL TMS versus sham during semantics showed significantly decreased deactivation in the semantic network. (D) TMS versus sham contrasts during social cognition showed increased deactivation in aDMN after right IPL TMS and in pDMN-IPL after TMS in both hemispheres. Results are reported for modulations with p < 0.05 of 10,000 permutations (asterisks).

Using the time series of ICA networks from all TMS conditions, task-specific network activity was assessed at baseline and then tested for stimulation effects. To this end, stimulus condition-specific responses were estimated for each network through multiple temporal regression analyses with the GLM design matrices. We identified task-active networks at baseline with pairwise tests between sham condition target and control responses (Fig. 9A, Table S5). After sham TMS, the number of task-active networks at baseline increased across domains, from attention to semantics to social cognition. During attentional reorienting the DAN, VAN, and rFPCN were significantly activated. During semantics, the semantic network, aDMN, DMN and VAN were activated while the DAN was deactivated. Social cognition recruited aDMN, DMN, pDMN-IPL, and the semantic network while deactivating DAN, VAN, rFPCN, CON, and the motor network.

Network-level TMS modulations of activity were identified through pairwise comparison of target-only responses of each network in sham and active TMS conditions. IPL stimulation effects on task activity occurred mainly in task-active networks (Fig. 9, Table S5). For attention, left IPL stimulation decreased rFPCN activity and aDMN deactivation (rFPCN: p = 0.0293, aDMN: p = 0.0302, Fig. 9B). Semantic network deactivation during semantic cognition was reduced after right IPL TMS (Fig. 9C). During social cognition, DMN subnetworks were further deactivated after TMS, specifically for pDMN-IPL after stimulation in either hemisphere (left: p = 0.0364, right: p = 0.0299) and aDMN after right-hemisphere stimulation (p = 0.0425, Fig. 9D). Subject-level target response betas for all TMS conditions across networks and tasks are displayed as supplementary information (Fig. S3).

### 2.7 TMS modulations of network interactions are state-specific

Domain-specific functional connectivity between networks was estimated for all TMS conditions using correlational psychophysiological interactions (cPPI) analysis and partial correlations of network time series for task and resting states, respectively. In the baseline condition, network interactions showed similar core architecture connectivity across domains (Fig. S4). Expected interactions included strong DAN-VAN correlation accompanied by both networks’ anticorrelation to aDMN during attention, strong coupling between the semantic network and other default mode subnetworks during semantic cognition, and more pairs of positively connected networks during social cognition, relative to attention and semantics. Resting-state partial correlations revealed generally weaker network pair interactions overall, relative to task-oriented cognition.

**Fig. 10:**
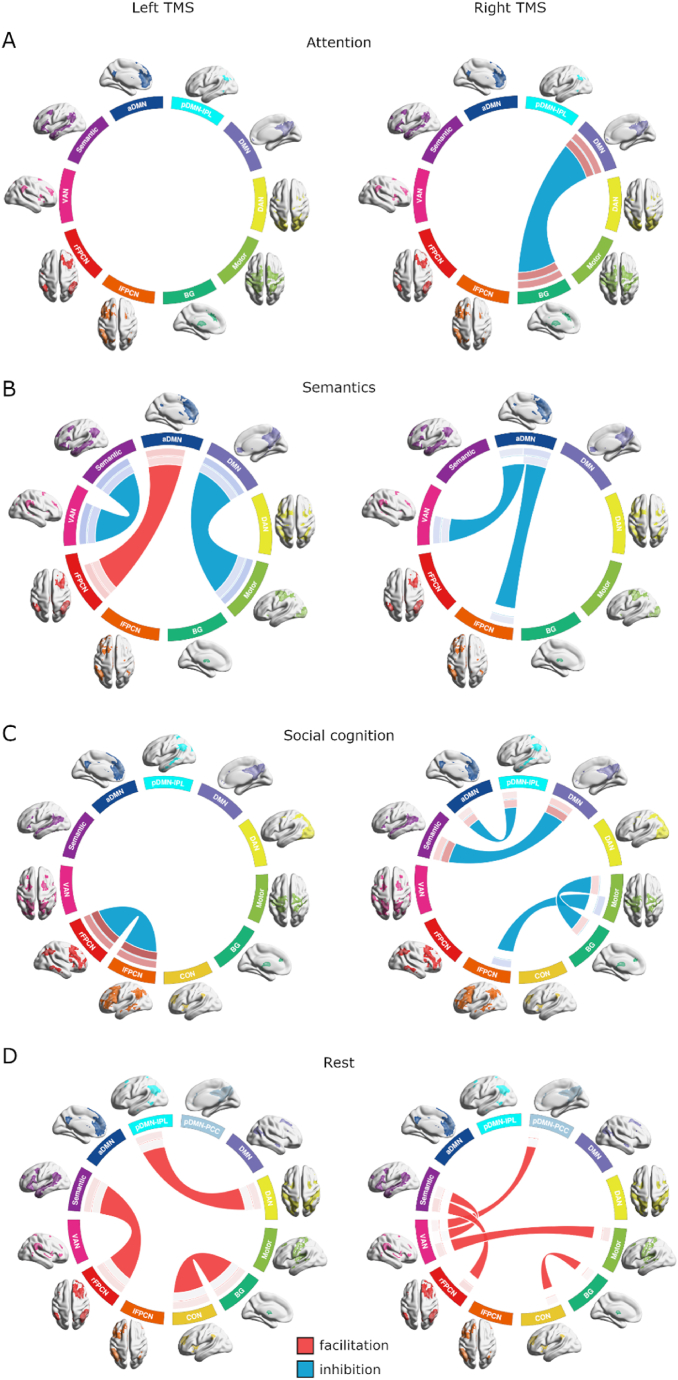
TMS modulates network interactions in a state-dependent manner. TMS effects on domain-specific network interactions revealed by contrasting between-network connectivity for active (left or right) TMS against sham within each task domain. Tests for resting state contrasted mean pre-post TMS connectivity differences in active and sham conditions. Significant network connectivity differences from sham for left and right TMS are illustrated in the left and right columns, respectively, for all domains. At the end of the red/blue connecting lines, inner and outer circle sections indicate correlation strength for baseline and active TMS, respectively (See Fig. S4 for baseline connectivity). (A) In attention, right TMS inhibited interaction between DMN and the BG network. (B) In semantics, left TMS modulated interactions between three pairs of networks, and right TMS inhibited aDMN interaction with VAN and lFPCN. (C) In social cognition, left TMS inhibited interaction between the two frontoparietal control networks, and right TMS inhibited interactions between four network pairs. (D) During rest, left and right TMS facilitated interactions between several network pairs. Results reported are comparisons with p < 0.05 in 10,000 permutations.

Within each domain, we tested for TMS-induced changes in connectivity between networks through pairwise comparisons of active and sham TMS conditions. A general pattern emerged from the TMS modulations such that IPL stimulation decreased interactions between networks during goal-oriented cognition and increased interactions during resting state (Fig. 10, Table S6). For attention, only right-hemisphere stimulation exhibited changes in network connectivity, specifically inhibition between the DMN and the basal ganglia network. In semantics, left IPL stimulation facilitated interaction between aDMN and rFPCN and otherwise inhibited interactions between the DMN and motor network and the VAN and semantic network. During social cognition, TMS inhibited several network pair interactions. Left and right FPCN coupling decreased after left-hemisphere stimulation. Right-hemisphere stimulation inhibited aDMN to pDMN-IPL, semantic network to DMN, and motor network to, each, BG network and lFPCN. Interestingly, during rest, only facilitation between networks occurred. After stimulation in either hemisphere, increased coupling occurred between the BG and cingulo-opercular networks and the semantic network and rFPCN. Left TMS also increased connectivity between pDMN-IPL and DAN, while right TMS facilitated VAN connectivity to pDMN-IPL, motor, and semantic networks.

## 3 Discussion

In this study, we employed offline TMS-fMRI to demonstrate regional and large-scale network adaptations to focal disruption of the left or right inferior parietal lobe, a key cognitive hub, in healthy humans. We aimed to shed light on whether network-level compensations occurring to uphold normal cognitive function are specific to the hemispheric functional specializations of the attention, semantic, and social cognition domains.

Our main findings were as follows: First, univariate analyses revealed that TMS-induced modulation of task-related activity varied more across domains than between the hemispheres perturbed, mainly resulting in facilitation in midline DMN regions during attention and inhibition in distributed areas during semantics and social cognition. With respect to regional changes in intrinsic functional connectivity, TMS over left IPL decreased connectivity in the extended target area and bilaterally across DAN regions, including the cerebellum, at rest. TMS over right IPL inhibited resting-state connectivity to within-hemisphere regions anterior to the target and mainly within the DAN. Hemisphere-specific IPL connectivity profiles that differed across the cognitive domains correspond to typical activations reflecting hemispheric specialization in each task. Large-scale networks also showed spatial distributions in connectivity specific to each domain. Accordingly, domain-specific network topography and laterality profiles of IPL connectivity distinguished left from right TMS effects. Regarding temporal dynamics of large-scale networks, TMS differentially affected network activity and between-network connectivity in a task-specific manner. We found that TMS mainly affected the activity of task-active networks, with a mixture of inhibitory and facilitatory effects, despite not impacting task performance. Furthermore, between-network connectivity changes after TMS differed between tasks and rest, indicating a state-specific response. At rest, network interactions were exclusively facilitated after left and right IPL stimulation. In contrast, task-related network interactions were mainly inhibited after TMS, both over left and right IPL. At the network level overall, TMS over IPL modulated activity and connectivity primarily among frontoparietal networks and default mode subnetworks. In the absence of behavioral perturbation effects, these changes in functional connectivity and neural activity likely represent adaptations required to sustain task performance.

In the following discussion, we integrate results across the range of analyses to summarize the dominant themes in our observations.

### 3.1 Cognitive domain shapes functional connectivity, influencing TMS effects

Regarding the influence of domain-specific FC on TMS effects, we first gained insight through characterizing connectivity to the stimulation targets for each cognitive domain. Directly comparing left and right IPL whole-brain FC highlights lateralization features across domains that likely contribute to hemisphere-specificity of TMS-induced modulations. We show that left and right IPL connectivity are the most similar and least lateralized during rest, paralleling similarities in left and right TMS responses, such as common between-network connectivity increases (Semantic-rFPCN and CON-BG). TMS over right IPL triggered more modulations than over left IPL in the right IPL-lateralized social cognition task. This was not the case for attention, which shows a stronger right-lateralization of IPL connectivity than social cognition. However, compared to social cognition, left and right IPL connectivity profiles are more similar in attention, during which TMS induced similar changes in posterior midline activity after left and right TMS. Among the tasks, left and right IPL connectivity are most similar in semantics, while whole-brain connectivity lateralizes stronger to the left IPL. This combination of features may account for the seemingly paradoxical modulation of semantic network activity after right IPL stimulation, such that perturbation of a node in the task-active network triggers an easy shift of resources within the network to the better-connected homologue relative to more costly shifts to other networks. Hemisphere-specific variations of parietal cortex connectivity have been described across domains using functional connectivity analyses [15,17,[34], [35], [36]] and demonstrated with TMS [10,29,31,37,38].

Additionally, we show that large-scale FC networks resolve differently across cognitive domains [[39], [40], [41]]. For example, during semantics, relative to the other domains, the default mode fractionated into the fewest subnetworks and the lFPCN occupied the least cortical space. These results likely relate to the strong left-hemisphere dominance of language-related networks and supporting roles of other domain general networks such as the multiple demand network in various aspects of semantic cognition [[42], [43], [44]]. Such topographical redistribution of connectivity to form domain-specific networks becomes particularly relevant when considering their spatial relationships to the TMS targets. Emphasizing the functional diversity of the IPL, we show that the extent of stimulation seed overlap with each network varies across domains. Presumably, this change in stimulation coverage would interact with network targeting in each domain. Our results demonstrate this topographic influence in combination with task-active network responses discussed in the previous section. For example, aDMN overlaps with stimulation targets the least during semantic cognition, and we detected no change in activity in the network for that domain. Likewise, the right stimulation seed overlap with rFPCN is very similar during attention and semantics, and both are greater than during social cognition. TMS modulated rFPCN activity in attention but not in semantics, wherein the network did not contribute in the sham condition. pDMN-IPL was not evoked as a distinct network during semantics, and while the left stimulation seed overlapped with it most during attention, this network's activity changed only in its task-active social cognition domain.

Throughout our results we see nominal similarities in network responses to left and right IPL stimulation, wherein modulations occur in the same direction relative to sham. While similar effects for left and right IPL stimulation are likely due primarily to their structural symmetry, functional connectivity and structural connectivity show discordant attributes across hemispheres [45], therefore hemispheric functional specialization shapes local and whole-brain responses to stimulation.

### 3.2 TMS modulates activity of task-active networks

We show that TMS over IPL induced task-specific changes in network activation. Generally, responsive networks in each domain were active in the unperturbed condition for the respective task, including the rFPCN in attention, the semantic network in semantics, and aDMN and pDMN-IPL in social cognition. The reduced deactivation in aDMN in attention after left IPL stimulation appears to be an exception to this pattern since that network was not active during attention after sham TMS. Nevertheless, the univariate map of activity during attention after sham (Fig. 3) shows task-specific deactivation in posterior midline regions intersecting with aDMN, indicating that task-specific involvement of the network may be obscured when generalizing its activity to a single time series. These results are supported by previous concurrent TMS-fMRI studies in which TMS selectively perturbed regions engaged during task performance as well as remote areas considered to be part of task-relevant networks [37,46,47]. Our task-dependent results show that the after-effects of TMS also rely on the ongoing function of brain networks, as biophysical modeling of collective brain network dynamics has indicated [48]. Previous work demonstrated distributed task-specific inhibition in task-active networks during semantic processing after inhibition of the IPL [31]. Increased activation in neighboring areas was also observed for lower-order functions that may reflect compensatory adaptive plasticity [31]. Another study found that TMS only affected deep but not shallow memory encoding, arguing for the impact of the current task state [49]. Collectively, the previous and present results show domain-specific modulations of task-active network responses, supporting that, in addition to being state specific, TMS effects are task specific.

In each of the results described above, TMS-induced activity modulations were not detected in the networks with maximal E-field hotspot overlap in the respective domains. Rather, activity changed in task-active networks with small IPL nodes relative to the rest of the network. Although the exact mechanisms are not completely understood, it is well established that TMS induces changes in remote regions in the network of the cortical target [24,25,50]. Some studies have demonstrated the behavioral relevance of such remote effects for different tasks [31,51]. This finding further illustrates TMS responses as selective modulation of domain-specific activity through network dynamics.

### 3.3 Network interactions respond to TMS in a state-dependent manner

We show that TMS over IPL primarily decreased interactions between networks during attention, semantics and social cognition, which contrasts with facilitated interactions during unconstrained rest. These results indicate a state-based response to TMS, which is largely characterized by opposite between-network connectivity changes in task and rest states. These contrasting brain responses across task-free and task-active states are in line with other studies reporting network responses to targeted stimulation of a single node. For example, TMS over a posterior parietal node decreased connectivity between its hippocampal network and nodes of other task-related networks during memory formation [32]. In resting-state, increased between-network connectivity has been detected after applying TMS in a pre-post design [52]. One concurrent TMS-fMRI study reported reduced connectivity between networks in a task compared to rest [47]. Our results contribute evidence for contrasting changes during the two states in the same set of participants.

During tasks, the functional modularity of the brain has been reported to be reduced compared to resting state [53,54]. Moreover, modularity structure, characterized by subnetworks with high within-network connectivity and low between-network connectivity, requires a balance of segregation and integration for healthy brain function [55,56]. Such network properties could be one explanation for the state-dependency in TMS responses that we find. During tasks, to sustain normal behavioral function (performance) after network perturbation, task-relevant networks may respond by further disengaging with one another to retain within-network connectivity, i.e., segregation-integration balance, necessary for cognitive demands [57]. Such specific network segregation may not be required to maintain task-free cognition. Other resting-state studies have detected within- and between-network connectivity increases after TMS [52,58]. Likewise, the resting-state network adaptations reported here reflect connectivity increases within the whole-brain network as opposed to particular task-relevant ones.

### 3.4 Flexibly dynamic networks are the most TMS-responsive across conditions

Across all cognitive domains at the network level, TMS consistently modulated the temporal dynamics, either activity or connectivity, of frontoparietal and default mode networks. The DMN is a domain-general network with multimodal hubs that contribute variably to a range of cognitive functions, illustrating its high capability for dynamic reallocation of resources [5,59,60]. While we specifically targeted a hub node of the DMN, it is noteworthy that frontoparietal networks persistently modified their behavior after TMS. Flexibility to support diverse, dynamic ongoing function is a prominent characteristic of frontoparietal networks [61,62], as is their role in cognitive control [61,63]. Frontoparietal networks have been distinguished as intermediary networks, readily switching connectivity or co-activation preference between default mode and task-active networks throughout cognition [[64], [65], [66]].

Bolstering the roles of frontoparietal and default mode subnetworks in dynamic reconfiguration of networks for cognition is the complex relationship between the two sets. Differentiated frontoparietal and default mode subnetworks exhibit parallel distribution throughout the cortex and display preferential activation and coupling depending on factors such as task difficulty, context, and temporal window [[66], [67], [68], [69]]. It has been suggested that parallel subnetwork organization could increase functional resiliency in cases of perturbation or impairment [70]. Our finding of frontoparietal and default mode networks being universally affected by TMS is in line with the role of these domain-general networks in flexibly redistributing resources in response to continuously varying demands, including inhibition of a functional hub.

A limitation of our study is the use of uncorrected thresholds for some of the statistics of TMS effects. The overall weak effects likely demonstrate the burden of a node's inhibition being shared collectively as complex and subtle adjustments across networks [71]. Due to weak and variable responses to TMS, some prior TMS studies have reported weak or uncorrected results after null results in initial analyses [26,52]. This manner of reporting may provide the field with observable trends that could help guide future TMS studies. Furthermore, while we expect the effects of TMS to last at least 30–45 min, it is not known whether such effects slowly decay or have an “on-off” form. To avoid systematic influences of potential decay effects on a specific task, we counterbalanced the order of tasks across participants. To minimize variance related to the decay of TMS effects, we maintained the order of tasks within participants, such that TMS conditions could be fairly compared in pairwise tests (e.g., left vs. sham TMS) for each task. This decision allowed us to compare TMS modulations across the tasks within the effective time window, however it could also explain some of the weak effects we found at the group level. Nonetheless, we consider our results to collectively contribute to the understanding of TMS effects in human brain networks.

In conclusion, this study provides evidence that short-term perturbation of a hub node with TMS triggers state-specific alterations between flexible, domain-general networks and activity modulations in networks that contribute to domain-specific function, which in turn maintain ongoing cognitive processes.

## 4 Resource availability

### 4.1 Data and code availability

All data reported in this paper will be provided upon request to the corresponding author and original code is publicly available (https://github.com/kathleen-williams/network_adaptations_to_IPL_inhibition).

## 5 Methods

### 5.1 Experimental model and study participant details

We present data from 30 healthy, native German speakers (13 females, mean age 29 ± 4.8 years), who took part in the entire offline TMS-fMRI experiment. All participants were right-handed according to the Edinburgh Handedness Inventory [72] (laterality quotient >80 %), had normal hearing and normal or corrected-to-normal vision, and no history of neurological or psychiatric disorders or any contraindications against MRI. Participants were recruited from the in-house database at the Max Planck Institute for Human Cognitive and Brain Sciences. The experiment consisted of up to seven sessions for each participant with an optional initial resting motor threshold or structural MRI measurement for cases in which a participant's data was not already in the institute database. All subjects participated in six TMS-fMRI sessions separated at least one week apart to avoid repetition effects (Fig. 1). All participants gave written informed consent to participate in the experiment approved by the Ethics Committee of the Medical Faculty of the University of Leipzig, Germany (282/16-ek) and were compensated at 10 euros per hour. All the work described here was carried out in accordance with The Code of Ethics of the World Medical Association (Declaration of Helsinki) for experiments involving humans. Due to excessive motion in at least one task run (FD > 5), two participants were excluded from all task-based analyses (n = 28, task; n = 30, rest).

### 5.2 Method details

#### 5.2.1 Image acquisition

Data acquisition was performed on a 3 T S Skyra fit MR system (Siemens). For every blood-oxygen-level-dependent (BOLD) run, a 10-min (1189 vol) whole-brain gradient echo planar (GE-EPI) T2∗-sensitive sequence (3 × 3 × 3.2 mm, 0.32 mm gap, 0.5 s TR, 36 slices, 24 ms TE, 45° flip angle) with multiband acceleration was used [73]. Additionally, in a separate scanning session, two high-resolution (1 × 1 × 1 mm voxel size) structural MR images (T1- and T2-weighted) and one diffusion-weighted image set were acquired for each participant using a standard 3D magnetization-prepared rapid acquisition with gradient echo sequence. The T1-weighted image was used for neuronavigation, and all structural images were used for modeling the TMS-induced electric fields. An eye-tracking camera was used to allow experimenters to monitor participants during the scans and note occasions of sleep or drowsiness.

#### 5.2.2 fMRI experiment

The fMRI experiment included three resting-state sessions, which consisted of a pre-stimulation measurement and three post-stimulation measurements (10 min × 4 runs) and three task-based sessions that were performed after TMS. Each task fMRI session included three runs representing the cognitive domains of attention, semantics, and social cognition (10 min × 3 tasks). For both rest and task fMRI sessions, three stimulation conditions, including left IPL TMS, right IPL TMS, or sham TMS, were counterbalanced in order across participants, thus applying all stimulation conditions in all subjects. Stimuli, displayed and triggered using Presentation software (Version 16.3.x) [74], were projected onto a panel positioned at the end of the MRI bore, and a head coil-mounted mirror was positioned to center the projection in the participant's field of view. For task fMRI, the order of tasks was counterbalanced across participants but fixed within participant. Participants responded as quickly as possible via a two-finger button box placed in the right hand. The participants underwent task training prior to the stimulation procedure.

For resting-state, also referred to as “rest” or “task free,” measurements, a white crosshair centered on a black screen was presented to participants. Participants were instructed to visually focus on the cross, stay awake and as still as possible. For task fMRI, following our previous study, we used three well-established cognitive tasks that rely on intact IPL function and exemplify a range of larger cognitive domains [17]. A Posner-like reorientation task represented attention, lexical decisions typified semantics, and communicative intention processing evoked social cognition [[75], [76], [77]]. Trials were presented in an event-related fashion, and each task had a target condition and a well-matched control condition to be contrasted as a means of capturing domain-specific brain activity. Briefly, during the attentional reorienting task, a directional arrow appeared at the center of the screen in each trial to indicate, congruently or incongruently, the position of a visual target presented on the following screen, thereby directing the participant's attention to the left or right. In the invalid condition of interest, or target condition, the direction of the arrow was incongruent with the position of the target's appearance, forcing participants to reorient their attention (20 % of trials). Congruently appearing targets represented the valid control condition in 75 % of the trials. Catch trials in which no target appeared, therefore requiring no response, were also included (5 % of trials). In the lexical decision task, participants made decisions (word or pseudoword?) about a visually presented word (target condition) or pseudoword (control condition) based on concrete German nouns and well-matched pseudowords. In the social cognition task, comic strips of four panels were sequentially presented to participants, who were instructed to demonstrate their comprehension of stories by selecting logical story endings from two choices. The target condition, which represented prototypical social interaction, consisted of stories categorized conceptually as extralinguistic communicative intention, depicted by people making communicative gestures, such as pointing to a bottle to request it. The stories presented in the control condition belonged to the conceptual category of physical causality among objects, such as a ball blown by a gust of wind that knocks over and breaks several bottles.

#### 5.2.3 Stimulation and E-field computation

The cortical targets within both IPLs were based on a previous group study with the same attention and semantic tasks [17]. We targeted the group peak transformed into individual space (SPM) within the left IPL of the word-pseudoword contrast (MNI coord. x, y, z = −51, −72, 28) and the group peak within the right IPL of the invalid-valid contrast (MNI coord. x, y, z = 57, −48, 24) [78]. If necessary, the cortical target was shifted to the closest gray matter location. We constructed high-resolution head models from T1- and T2-weighted MRI scans and calculated the optimal coil position and orientation to stimulate the cortical targets with the simnibs.TMSoptimize(.) method. For the electric field simulation, gray and white matter anisotropy based on a DWI scan were included, and standard conductivity values were used for the five tissue types gray matter, white matter, cerebrospinal fluid, bone, and skin. These optimal coil configurations provided us with individual FEM-based E-field simulations. To allow for group analyses, we transformed the E-fields into common spaces. To optimize the cortical field exposure, we selected the coil position and orientation with respect to the maximum overall stimulation strength |E|, that is, the magnitude of E, in a 5 mm sphere around the cortical target, restricted to gray matter only [79]. Optimal coil positions and orientations from the optimization procedure were exported and imported into the neuronavigation software with the IMporter tool [80]. The rMT for each subject was assessed based on the 5-10-Rothwell [81] method, starting with individualized M1 targets based on Mayka et al. [82].

After neuronavigation and a short practice block, two cTBS600 trains [27] were delivered at 90 % resting motor threshold with a 10-min pause [83,84] to assure robust stimulation effects throughout the fMRI acquisition after TMS. Because TMS effects potentially interact with cognitive state [85,86], subjects watched a soundless “Inscapes” video during the 10-min break between cTBS trains [87]. TMS was delivered with a figure-of-eight coil (MagVenture MCF-B65 for the active TMS conditions ‘left IPL’ and ‘right IPL’; MagVenture MCF-P-B65 placebo coil in the ‘sham TMS’ condition over the left IPL target) connected to an ×100 stimulator. Coil positioning was guided by a neuronavigation system (TMS Navigator, Localite). Subjects were transferred to the MRI scanner immediately after TMS in an MRI-compatible wheelchair to minimize self-inflicted movement [88]. The entire stimulation and transfer procedure were conducted silently, and the time between the end of the second stimulation and the start of the fMRI measurement was below 10 min.

#### 5.2.4 Behavioral analysis

To test for TMS effects on task performance, correct response times and accuracies for attention, semantics, and social cognition were analyzed using paired t-tests between control and target conditions within domain and between sham and active target-only conditions to test for stimulation effects.

#### 5.2.5 Image preprocessing

FMRI preprocessing was implemented with fMRIPrep 21.0.1 [89], which is based on Nipype 1.6.1 [90] and Nilearn 0.8.1 [91]. A fieldmap was estimated based on two EPI references with topup (FSL 6.0.5.1) [92]. The T1-weighted image was corrected for intensity non-uniformity with N4BiasFieldCorrection [93,94] and skull-stripped with a Nipype implementation of the antsBrainExtraction.sh workflow, using OASIS30ANTs as the target template. Brain tissue segmentation of cerebrospinal fluid (CSF), white matter (WM), and gray matter (GM) was performed on the brain-extracted T1w using fast (FSL 6.0.5.1) [95]. Brain surfaces were reconstructed using recon-all (FreeSurfer 6.0.1) [96], and the brain mask was refined with a custom variation of the method to reconcile ANTs-derived and FreeSurfer-derived segmentations of the cortical gray-matter of Mindboggle [97]. Volume-based spatial normalization to the MNI152Lin template was performed with antsRegistration (ANTs 2.3.3). For each BOLD run a reference volume and its skull-stripped version were generated. Subsequently, head-motion parameters, i.e., transformation matrices and six rotation and translation parameters, were estimated using mcflirt (FSL 6.0.5.1) [98]. The estimated fieldmap was then aligned with rigid-registration to the target EPI reference volume and the field coefficients were mapped onto the reference EPI. BOLD runs were slice-time corrected to 0.214s using 3dTshift from AFNI [99,100]. Finally, the BOLD reference was co-registered with six degrees of freedom to the T1w reference using bbregister (FreeSurfer) which implements boundary-based registration [101]. Framewise displacement (FD) was computed as a time-series confound using the absolute sum of relative motion formulation following Power [102]. Additionally, a set of physiological regressors were extracted to allow for component-based noise correction (CompCor) [103]. Principal components were estimated after high-pass filtering the preprocessed BOLD time series (using a discrete cosine filter with 128 s cut-off) for the anatomical CompCor variant (aCompCor). Three probabilistic masks (cerebral spinal fluid, CSF; white matter, WM; and combined CSF + WM) were generated in anatomical space, following the fmriPrep implementation. Components are also calculated separately within the WM and CSF masks. The confound time series derived from head motion (x, y, z, yaw, pitch, roll) estimates were expanded with the inclusion of temporal derivatives and quadratic terms for each, yielding 24 total motion parameters [104]. Details of the pipeline can be found in fMRIPrep workflows (https://fmriprep.org/en/stable/workflows.html). For restricting later analyses and statistical tests to voxels of interest, binary gray-matter masks were generated from the SPM12 tissue probability map (probability >0.3). Preprocessed data were smoothed with a 4 mm FWHM 3D Gaussian kernel.

#### 5.2.6 Voxelwise analyses

##### 5.2.6.1 fMRI general linear model

Task fMRI data were analyzed using the standard two-level mass-univariate GLM approach in SPM12. First-level analysis was initiated by constructing GLMs for each participant run using the FAST(1) algorithm to account for temporal autocorrelation in fMRI data with short repetition times [105]. The model included a regressor for each stimulus condition (i.e., target and control) modeling the onset and duration of every correct trial stimulus in an fMRI run. Additionally, it included nuisance regressors for steady-state outliers, 24 motion parameters, the first six aCompCor and six corresponding cosine regressors, FD, and individual regressors for censoring each time point with excessive volume-to-volume movement (FD > 0.5). For all group-level voxelwise analyses, a gray-matter mask was applied to restrict statistical tests to relevant voxels for random-effects t-tests. To characterize baseline task activity in the brain, target-versus-control contrast images were computed for each participant and all tasks, and one-sided t-tests were performed and statistically thresholded at a voxelwise p < 0.001 and a cluster-level p < 0.05, corrected for multiple comparisons using the family-wise error (FWE) method. To test for modulatory TMS effects, contrast images were computed by modeling the target against implicit rest to avoid interaction effects of the task conditions, and target-only contrast images were submitted to paired t-tests between active (left or right) and sham stimulation with results thresholded to p < 0.05 in the voxel, and p < 0.05, FWE-corrected at the cluster level.

##### 5.2.6.2 Seed-based functional connectivity

We employed seed-based functional connectivity analyses to investigate how TMS modulations relate to IPL's functional connectivity with the whole brain across cognitive domains. To this end, voxelwise analyses were carried out using seeds derived from the E-field simulations. Subject-specific seed ROIs were generated from E-field models of left and right IPL stimulation. The E-fields in MNI space were cytoarchitectonically constrained to IPL, defined according to the JuBrain probabilistic cytoarchitectonic atlas [17,106]. Anatomically defined, IPL in each hemisphere comprised seven regions, which were provided by the SPM 12 Anatomy Toolbox v2.2 b [107]: rostral and caudal angular gyrus (PGa and PGp, respectively), and five regions of the supramarginal gyrus (PFm, PF, PFop, PGcm, PFt). E-fields were thresholded to the 90th percentile of their maximum magnitude (|E|) [108] and binarized. Group-level representations of E-field hotspots were generated by summing the binary seed maps across participants for each hemisphere. The group-level maps were binarized and used to estimate stimulation field overlap with large-scale networks.

Intrinsic FC was estimated using the resting-state data in a simple cross-correlation analysis. In detail, data was denoised prior to analysis through temporal regression of the same nuisance parameters included in the univariate analysis, then AFNI's 3dRSFC function was employed to bandpass filter data (0.01–0.1 Hz). Note that we did not perform global signal regression for any resting-state data analyses to ensure that processing is matched as similarly as possible to task-based analyses. However, including aCompCor regressors in the set of nuisance variables, robustly removes much of the noise associated with the global signal from connectivity measures [109]. For each hemisphere, the mean time series of the subject-specific seed was cross-correlated with that of each voxel in the brain, yielding a statistical map that was Fisher's Z-transformed for second-level analyses. This procedure was applied to every resting-state fMRI run in the experiment, yielding subject- and session-level intrinsic FC maps for left and right IPL that were submitted to voxelwise group-level statistics. To characterize baseline IPL intrinsic FC at the group level, pre-stimulation resting-state seed-based connectivity maps, averaged within participant across experimental sessions, were submitted to one-sided t-tests. To capture modulatory TMS effects, we conducted paired t-tests between active (left or right) and sham stimulation difference maps (average of post-TMS - pre-TMS). For the within-condition averages, we excluded difference maps derived from runs in which maximum FD > 1. Baseline maps were statistically thresholded at a voxelwise p < 0.001 and a cluster-level p < 0.05, corrected for multiple comparisons using the family-wise error (FWE) method. For TMS effects, results were thresholded at p < 0.05 in the voxel, and p < 0.05, FWE-corrected at the cluster level. Pre-stimulation FC maps were additionally used to calculate indices of hemisphere-specific connectivity at rest.

To probe task-specific FC from the stimulated regions of the IPL to the whole brain, we employed the gPPI toolbox in SPM12 to characterize voxelwise task-dependent FC to a seed ROI, independent of intrinsic correlations and task-related activation [110,111] In each task, for both hemisphere ROIs separately, we performed whole-brain analysis based on the GLM. Participant data were modeled separately at the first level, using the participant-level GLMs generated for the univariate analysis as the basis for the gPPI analysis. The psychological regressors modeled the contrast of correct trials of the target and control experimental conditions, the physiological regressors were composed of the first eigenvariate of the seed ROI time series, and PPI regressors for each experimental condition were created through multiplication of the HRF-deconvolved seed ROI BOLD signal with the condition onsets, followed by convolution with the canonical HRF [110,112]. The model also included the nuisance regressors that were used for univariate analysis. For each hemisphere and within each task, contrast images were computed for each participant and used to calculate indices of hemisphere-specific connectivity in the tasks.

##### 5.2.6.3 Hemisphere-specific connectivity across domains

To inform potential underlying mechanisms of hemisphere-specific stimulation effects, we directly compared whole-brain seed-based connectivity of left and right IPL for each cognitive domain. For each subject, left IPL FC maps were correlated by volume to right IPL FC maps, without threshold, for attention, semantics, social cognition, and rest (AFNI 3ddot). In addition, laterality indices were computed for each subject in each domain. Laterality was calculated first at the voxel level by subtracting right IPL FC from left IPL FC and normalizing by the sum of the absolute values of left and right IPL FC [113]. Then, domain-wise laterality indices were summarized for each subject accordingly by averaging the voxel-level indices within a gray-matter mask. Group-level means and standard deviations for each domain initially included all participant summary values, then means were calculated excluding outliers that exceeded a threshold of three standard deviations away from the initial group mean.

#### 5.2.7 Network analyses

##### 5.2.7.1 Spatial ICA

To identify task-specific networks in a data-driven manner, groupwise spatial ICA and group information guided back reconstruction was performed separately for each cognitive domain, including resting state, using the GIFT toolbox (GroupICAT v4.0d) [114,115]. Only sham stimulation sessions for tasks, or three pre-stimulation sessions for rest, were included in ICA. In detail, preprocessed, smoothed data were initially intensity-normalized before data dimensions were reduced to 30 dimensions in each step of a two-step principal component analysis (PCA) procedure, with PCA performed initially at the session level, then concatenated for group-level PCA, reducing the time dimension to 30 for the whole group, giving the number of independent components extracted. Icasso repeated ICA 50 times to estimate the stability of the components [116]. Components were scaled to Z-scores within each component for the remaining analyses. Before reconstructing the resulting independent components in each task, noise and artifact components were identified for exclusion using visual inspection. As a general guideline, the highest Z-score voxels show the regions with the greatest contribution to a component's corresponding time series, and components with z-score peaks located outside gray matter were classified as artifacts and excluded from further analysis [117]. The group-level components classified as cortical sources were used as spatial templates for image-guided back-reconstruction of each component to sham, or pre-stimulation sessions as well as active stimulation sessions using group information guided ICA implemented in GIFT [115]. Group-level statistical maps of ICA networks were obtained for each domain by submitting all participant sham (task) or pre-stimulation (rest) session spatial maps to a one-sided *t*-test using a gray-matter mask, and thresholding on the voxel level at p < 0.001 and cluster-level p < 0.05, FWE-corrected. Thresholded maps were binarized to generate masks used for identification, visualization, and quantifying overlap with electric field hotspots.

##### 5.2.7.2 Network identification

The non-noise components were labeled and selected for networks of interest. Cortical ICA networks excluded from temporal analysis comprised low-level domain-specific networks, such as primary visual, auditory, and somatosensory networks. To match network components across cognitive states and relate them to well-known cognitive networks, AFNI's 3dMatch was utilized on group-level maps to calculate weighted 3D correlations and sort each task's selected component set, referencing them each to the set of resting state-derived networks and, separately, a set of templates of cortical networks recognized in the literature. The 3dMatch function calculates weighted 3D correlations of statistical maps to quantify the spatial similarity between pairs of brain maps in different sets and uses the value to assign the best-matching pairs. Literature-based templates were chosen for their relevance in analytical technique or experimental design. Intensity-based result template maps included 10 ICA-derived networks whose maps were well matched with cognitive profiles in the BrainMap database [118] and the ALE-derived general semantic cognition network described by Jackson and colleagues [119]. They were thresholded to positive voxels with a value of at least 20 % of the maximum value in the volume. Additionally, the template battery included binary masks of each network from the 17-network parcellation reported by Yeo and colleagues [120]. All template maps were resampled to the resolution of the BOLD data before calculating similarity indices. Network correlations to literature-based templates were only used for guidance in network labeling and are not reported here.

##### 5.2.7.3 Spatial overlap between stimulation seeds and networks

We overlapped stimulated regions onto the large-scale networks to elucidate which networks were most targeted directly by TMS. For each domain, group-level E-field hotspot maps were binarized and overlaid on network masks (Displayed in Fig. S2), then voxelwise overlap was quantified for each hemisphere-network pair as a percentage of the network. These quantities capture a spatial aspect of functional network reconfiguration occurring across domains and how that relates to the stimulated regions.

##### 5.2.7.4 Network activity

Multiple regression analyses estimated each network's activity during the three task-based fMRI sessions under all stimulation conditions. Using the GLM design matrices constructed for the voxelwise analyses, the *temporal sorting* utility in GIFT performed regression analyses between the network time series and the experimental stimuli at the subject level. The regression step yielded task predictor-specific beta values for each component, representing network activity for each task's target and control condition in all experimental sessions. Pairwise comparisons of activity betas were made for all tasks to classify which networks exhibit task-specific activation and which networks modulated activity after TMS. For each network, betas for sham sessions were used to perform pairwise permutation testing (n = 10,000, p < 0.05, Bonferroni-Holm multiple comparisons corrected) between each task's target and control conditions. To detect influences of TMS on task-specific network activity, pairwise permutation testing was applied in each task for each network between target betas of active (left or right) and sham stimulation conditions (n = 10,000, p < 0.05). For permutation testing, we implemented the following procedure in R: First, T_obs_ was computed as the mean beta difference between the tested conditions (i.e., target-control for sham activity and target active-sham for TMS) across subjects. Second, a permutation-based baseline distribution (10,000 values) T∗ was computed by shuffling the condition factor (e.g., ‘active’ or ‘sham’) for each subject before computing the average beta difference. Third, significance was reached if T_obs_ was more extreme than 95 % of T∗.

##### 5.2.7.5 Network connectivity

Connectivity between networks was estimated for all cognitive states. As in our previous study, we used correlational PPI (cPPI) to assess task-specific network connectivity [121]. Network time series from all runs were denoised prior to connectivity analyses through regression of the same nuisance time courses that were used for the voxelwise analyses. cPPI analysis, which we implemented using a freely available toolbox provided by Fornito and colleagues, estimates pairwise interactions between all regions, or networks, using partial correlations [122]. On the session level, for every pairwise interaction, two PPI terms were generated with the contrast between the target and control conditions of the task and the denoised ICA output time series from each of two networks of interest. A partial correlation was performed between the two PPI terms, while controlling for the activity of the remaining networks. Explicitly, we contrasted invalid versus valid for attention, word versus pseudoword for semantics, and communicative intention versus physical causality for social cognition. Between-network connectivity was estimated for resting-state runs by computing pairwise partial correlations of network time series that partialled out the signals of the remaining networks. Thus, network connectivity matrices were computed for all sessions, resulting in 21 (attention, semantics, social cognition, pre-stimulation rest, and three post-stimulation rest across three stimulation conditions) symmetric correlation matrices for each subject. Connectivity values of each network pair were tested for stimulation effects at the group level with pairwise permutation tests conducted between active (left or right) and sham stimulation conditions (n = 10,000). For the resting-state experiments, pre-stimulation connectivity matrices were used as a baseline and subtracted from each post-stimulation connectivity matrix at the session level. Second-level tests were conducted on the average of the three difference matrices. Results were considered significant at p < 0.05.

## CRediT authorship contribution statement

**Kathleen A. Williams:** Writing – review & editing, Writing – original draft, Visualization, Methodology, Investigation, Formal analysis, Data curation. **Ole Numssen:** Writing – review & editing, Software, Investigation, Formal analysis, Data curation. **Juan David Guerra:** Writing – review & editing, Formal analysis. **Jakub Kopal:** Writing – review & editing, Supervision. **Danilo Bzdok:** Writing – review & editing, Funding acquisition, Conceptualization. **Gesa Hartwigsen:** Writing – review & editing, Supervision, Project administration, Funding acquisition, Conceptualization.

## Declaration of competing interest

The authors declare that they have no known competing financial interests or personal relationships that could have appeared to influence the work reported in this paper.
