# Supplementary material for: Inhibition of the inferior parietal lobe triggers state-dependent network adaptations
Source: Heliyon. 2024 Oct 23;10(21):e39735. doi: 10.1016/j.heliyon.2024.e39735 (PMC11570486; doi:10.1016/j.heliyon.2024.e39735)
Supplement: Multimedia component 1 [file mmc1.docx]

# S1. Supplemental information


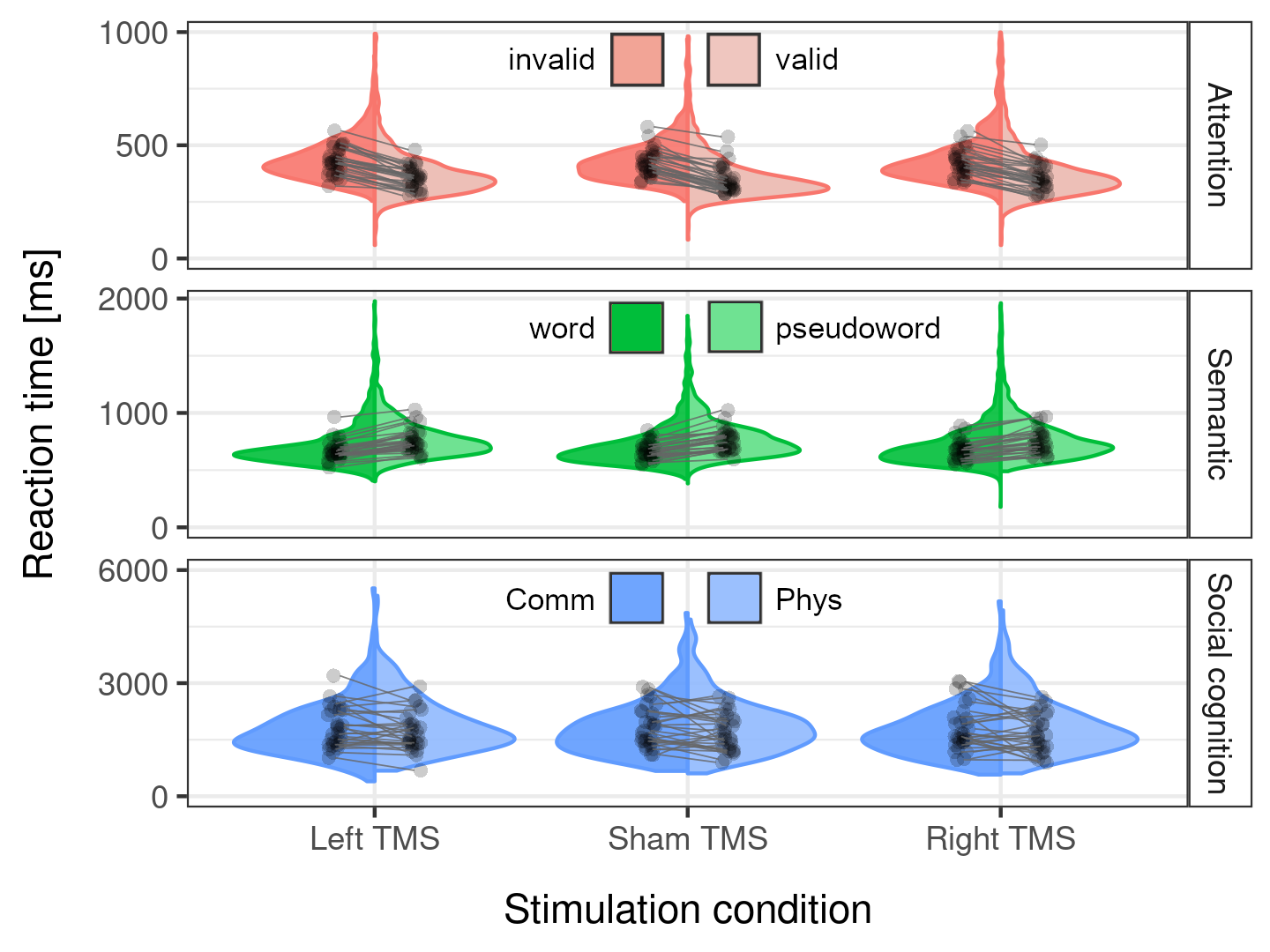


FIGURE S1: TMS did not affect behavior in any task. Behavioral results violin plots show reaction times in milliseconds for attention, semantics, and social cognition tasks in all stimulation conditions. Participant averages are plotted in gray. Comm.: communicative intent; Phys.: physical causality.

### TABLE S1: Behavioral Results

| Task | Trial type | Reaction time | | |  | Accuracy | | |
| --- | --- | --- | --- | --- | --- | --- | --- | --- |
|  |  | Left TMS | Sham TMS | Right TMS |  | Left TMS | Sham TMS | Right TMS |
| Attention | invalid | 436 ±90 | 428 ±89 | 436 ±107 |  | 97.50 | 95.83 | 96.53 |
|  | valid | 373 ±106 | 365 ±108 | 374 ±113 |  | 98.70 | 97.19 | 98.00 |
| Semantic | word | 781 ±186 | 785 ±186 | 717 ±188 |  | 95.83 | 95.06 | 96.15 |
|  | pseudoword | 706 ±177 | 706 ±171 | 779 ±196 |  | 96.43 | 94.46 | 96.15 |
| Social Cognition | Comm. | 1798 ±726 | 1879 ±745 | 1882 ±781 |  | 92.38 | 95.24 | 96.67 |
|  | Phys. C. | 1918 ±770 | 1793 ±713 | 1747 ±687 |  | 95.71 | 88.57 | 90.99 |

Notes: Reaction times are provided in mean ms ±SD ms, accuracy in %.

### TABLE S2: Univariate results – sham

| Significant clusters: sham univariate results | | | | | | | |
| --- | --- | --- | --- | --- | --- | --- | --- |
| # voxels in cluster | center of mass x | center of mass y | center of mass z | Mean T | SEM T | Max T | Eickhoff-Zilles atlas locations |
| Attention: invalid > valid | | | | | | | |
| 348 | 36.3 | -61.7 | 43.8 | 4.14 | 0.03 | 6.72 | R superior parietal lobule, R inferior parietal lobule, R postcentral gyrus, R middle occipital gyrus, R superior occipital gyrus, R supramarginal gyrus, R angular gyrus, R precuneus |
| 270 | -36.9 | -57.9 | 45.2 | 4.31 | 0.04 | 6.59 | L inferior parietal lobule, L superior parietal lobule, L postcentral gyrus |
| 194 | 53.9 | -66.0 | -3.4 | 4.15 | 0.04 | 7.21 | R middle temporal gyrus, R inferior temporal gyrus, R middle occipital gyrus, R superior temporal gyrus, R inferior occipital gyrus |
| 84 | 3.2 | -62.6 | 50.8 | 4.35 | 0.08 | 6.70 | R precuneus, L precuneus, R paracentral lobule, R postcentral gyrus, R superior parietal lobule |
| 44 | 31.5 | -7.4 | 59.9 | 4.21 | 0.11 | 6.44 | R middle frontal gyrus, R superior frontal gyrus |
| 41 | -33.2 | -11.2 | 55.6 | 4.14 | 0.08 | 5.56 | L middle frontal gyrus, L precentral gyrus, L superior frontal gyrus |
| 35 | 63.1 | -44.8 | 28.8 | 3.93 | 0.05 | 4.64 | R supramarginal gyrus, R inferior parietal lobule, R superior temporal gyrus |
| 29 | -54.3 | -67.5 | -11.4 | 3.95 | 0.08 | 5.36 | L middle temporal gyrus, L inferior temporal gyrus, L inferior occipital gyrus, L middle occipital gyrus |
| 26 | 3.0 | 43.3 | -11.0 | -3.78 | 0.06 | -4.55 | R rectal gyrus, L rectal gyrus, R mid orbital gyrus, R superior orbital gyrus, L mid orbital gyrus |
| 25 | -37.1 | 16.3 | 4.2 | 4.08 | 0.14 | 5.61 | L insula lobe, L inferior frontal gyrus (pars triangularis), L inferior frontal gyrus (pars orbitalis) |
| 24 | -50.9 | -81.4 | 2.0 | 3.98 | 0.10 | 5.58 | L middle temporal gyrus, L middle occipital gyrus |
| 23 | 25.3 | -81.8 | -21.9 | 4.08 | 0.11 | 5.49 | R fusiform gyrus, R lingual gyrus, R inferior occipital gyrus, R cerebellum (vi) |
| 22 | -30.2 | -82.1 | 22.2 | 3.79 | 0.06 | 4.47 | L middle occipital gyrus, L inferior parietal lobule, L superior occipital gyrus, L superior parietal lobule |
| 21 | 46.8 | 2.8 | 33.5 | 3.80 | 0.07 | 4.78 | R inferior frontal gyrus (pars opercularis), R precentral gyrus, R middle frontal gyrus, R inferior frontal gyrus (pars triangularis) |
| 19 | -2.6 | -71.2 | 17.1 | -3.78 | 0.05 | -4.20 | L cuneus, L precuneus, R precuneus, L calcarine gyrus |
| 18 | -50.8 | -1.4 | 32.0 | 3.74 | 0.07 | 4.32 | L precentral gyrus, L inferior frontal gyrus (pars opercularis), L inferior frontal gyrus (pars triangularis) |
| 16 | 33.3 | 19.6 | 6.9 | 4.31 | 0.15 | 5.74 | R insula lobe, R inferior frontal gyrus (pars triangularis) |
| 13 | 54.3 | -46.6 | 15.4 | 3.87 | 0.12 | 4.63 | R superior temporal gyrus, R supramarginal gyrus, R middle temporal gyrus |
| Semantics: word > pseudoword | | | | | | | |
| 858 | -1.8 | -44.7 | 38.4 | 4.37 | 0.03 | 7.99 | L precuneus, L middle cingulate cortex, R precuneus, R middle cingulate cortex, R paracentral lobule, R SMA, L paracentral lobule, L SMA, L cuneus, L posterior cingulate cortex, L calcarine gyrus, R calcarine gyrus, L anterior cingulate cortex, L superior parietal lobule, R posterior cingulate cortex, R postcentral gyrus, R anterior cingulate cortex, L postcentral gyrus, R cuneus |
| 418 | 60.3 | -43.5 | 16.1 | 4.22 | 0.03 | 6.59 | R supramarginal gyrus, R superior temporal gyrus, R middle temporal gyrus, R angular gyrus, R rolandic operculum, R inferior parietal lobule, R postcentral gyrus |
| 275 | -53.4 | -69.3 | 16.9 | 4.20 | 0.04 | 7.06 | L angular gyrus, L middle temporal gyrus, L supramarginal gyrus, L middle occipital gyrus, L inferior parietal lobule, L superior temporal gyrus |
| 191 | -49.6 | 5.5 | 25.3 | -4.42 | 0.06 | -7.11 | L inferior frontal gyrus (pars opercularis), L inferior frontal gyrus (pars triangularis), L precentral gyrus, L middle frontal gyrus, L temporal pole, L rolandic operculum |
| 129 | -62.1 | -13.2 | -27.3 | 4.29 | 0.06 | 7.09 | L middle temporal gyrus, L inferior temporal gyrus |
| 105 | -56.1 | -18.7 | 5.1 | 4.05 | 0.05 | 5.66 | L superior temporal gyrus, L rolandic operculum, L supramarginal gyrus, L postcentral gyrus, L insula lobe, L middle temporal gyrus, L temporal pole |
| 84 | -3.6 | 10.7 | 56.5 | -4.29 | 0.07 | -6.14 | L SMA, L superior medial gyrus, R superior medial gyrus, R SMA, L superior frontal gyrus |
| 76 | 4.8 | 51.0 | -0.9 | 4.17 | 0.06 | 6.25 | R mid orbital gyrus, R rectal gyrus, R superior medial gyrus, L mid orbital gyrus, L rectal gyrus, L superior medial gyrus, R superior orbital gyrus |
| 75 | 62.4 | -9.5 | -24.2 | 4.05 | 0.06 | 5.87 | R middle temporal gyrus, R inferior temporal gyrus, R medial temporal pole |
| 67 | -7.4 | 56.8 | 17.6 | 4.07 | 0.05 | 5.08 | L superior medial gyrus, L mid orbital gyrus, L superior frontal gyrus, L anterior cingulate cortex, L superior orbital gyrus |
| 55 | 49.4 | -77.7 | 21.9 | 4.07 | 0.07 | 5.71 | R angular gyrus, R middle occipital gyrus, R middle temporal gyrus, R inferior parietal lobule |
| 48 | 57.2 | -1.4 | 0.7 | 3.89 | 0.04 | 4.59 | R temporal pole, R superior temporal gyrus, R rolandic operculum, R insula lobe, R Heschls gyrus, R inferior frontal gyrus (pars opercularis) |
| 46 | 46.8 | 7.1 | 29.4 | -4.17 | 0.08 | -5.32 | R inferior frontal gyrus (pars opercularis), R inferior frontal gyrus (pars triangularis), R middle frontal gyrus |
| 38 | -33.9 | -61.6 | 45.6 | -3.89 | 0.07 | -4.93 | L superior parietal lobule, L inferior parietal lobule |
| 35 | 31.8 | -70.2 | 46.4 | -3.86 | 0.07 | -5.01 | R superior parietal lobule, R angular gyrus, R inferior parietal lobule |
| 33 | -13.3 | -103.3 | 8.1 | 4.04 | 0.11 | 5.77 | L superior occipital gyrus, L cuneus |
| 32 | -30.3 | -52.1 | 59.7 | 4.11 | 0.13 | 6.77 | L postcentral gyrus, L superior parietal lobule |
| 29 | -52.8 | -44.9 | 42.6 | -3.95 | 0.09 | -5.34 | L inferior parietal lobule, L postcentral gyrus |
| 28 | -39.6 | 24.3 | -3.4 | -3.97 | 0.09 | -5.47 | L inferior frontal gyrus (pars orbitalis), L insula lobe, L inferior frontal gyrus (pars triangularis) |
| 27 | 33.6 | -84.3 | -47.4 | 4.11 | 0.13 | 6.23 | R cerebellum (crus 2), R cerebellum (crus 1) |
| 26 | -30.2 | -25.7 | -24.0 | 4.30 | 0.13 | 5.94 | L parahippocampal gyrus, L fusiform gyrus, L hippocampus |
| 25 | -36.7 | 20.7 | 50.0 | 3.91 | 0.07 | 4.83 | L middle frontal gyrus |
| 21 | 51.1 | 32.1 | 23.9 | -3.94 | 0.08 | -4.84 | R inferior frontal gyrus (pars triangularis), R middle frontal gyrus |
| 20 | -27.8 | -50.4 | -21.3 | 3.82 | 0.08 | 4.76 | L fusiform gyrus, L cerebellum (vi), L cerebellum (iv-v), L lingual gyrus |
| 20 | 3.2 | 56.3 | 29.8 | 4.00 | 0.12 | 5.30 | R superior medial gyrus, L superior medial gyrus |
| 19 | 28.7 | -29.9 | -18.0 | 4.04 | 0.18 | 7.04 | R parahippocampal gyrus, R fusiform gyrus, R hippocampus, R cerebellum (iv-v) |
| 15 | -47.5 | -25.0 | 43.3 | 3.93 | 0.10 | 4.90 | L postcentral gyrus |
| 15 | 19.6 | -1.0 | 68.0 | 3.79 | 0.07 | 4.45 | R superior frontal gyrus, R SMA |
| 15 | -31.3 | -77.9 | 23.3 | -3.78 | 0.07 | -4.41 | L middle occipital gyrus, L superior occipital gyrus, L inferior parietal lobule |
| 14 | -23.4 | -81.0 | -26.1 | 3.77 | 0.08 | 4.59 | L lingual gyrus, L fusiform gyrus, L inferior occipital gyrus, L cerebellum (vi) |
| Social cognition: communicative intention > physical causality | | | | | | | |
| 934 | 55.1 | -37.3 | -3.7 | 4.99 | 0.04 | 8.58 | R middle temporal gyrus, R angular gyrus, R superior temporal gyrus, R medial temporal pole, R inferior temporal gyrus, R supramarginal gyrus, R middle occipital gyrus, R inferior parietal lobule, R inferior occipital gyrus |
| 892 | -56.5 | -44.1 | -2.1 | 4.82 | 0.04 | 10.33 | L middle temporal gyrus, L angular gyrus, L inferior temporal gyrus, L middle occipital gyrus, L supramarginal gyrus, L medial temporal pole, L superior temporal gyrus, L inferior parietal lobule |
| 743 | 5.2 | -70.3 | -22.7 | -4.65 | 0.04 | -9.98 | R fusiform gyrus, L fusiform gyrus, R lingual gyrus, L calcarine gyrus, R cerebellum (vi), R calcarine gyrus, L lingual gyrus, L cerebellum (vi), L inferior occipital gyrus, R cerebellum (iv-v), R cerebellum (crus 1), R inferior occipital gyrus, L cerebellum (crus 1), L cerebellum (iv-v), L middle occipital gyrus, R cuneus, R paraHippocampal gyrus, L inferior temporal gyrus |
| 664 | 42.2 | -59.9 | 36.8 | -4.61 | 0.04 | -7.72 | R superior parietal lobule, R middle occipital gyrus, R inferior parietal lobule, R postcentral gyrus, R supramarginal gyrus, R superior occipital gyrus, R angular gyrus |
| 553 | -2.1 | -63.5 | 33.9 | 5.17 | 0.05 | 11.10 | L precuneus, R precuneus, L middle cingulate cortex, L posterior cingulate cortex, R middle cingulate cortex, R posterior cingulate cortex, L cuneus, L calcarine gyrus, L superior parietal lobule |
| 241 | -57.8 | -39.5 | 37.5 | -4.40 | 0.06 | -8.69 | L inferior parietal lobule, L supramarginal gyrus, L postcentral gyrus, L superior parietal lobule |
| 238 | -29.0 | -85.6 | 25.3 | -4.42 | 0.05 | -6.78 | L middle occipital gyrus, L superior parietal lobule, L inferior parietal lobule, L superior occipital gyrus, L precuneus |
| 139 | 59.1 | -54.7 | -21.9 | -4.66 | 0.08 | -9.13 | R inferior temporal gyrus, R middle temporal gyrus, R inferior occipital gyrus, R cerebellum (crus 1), R fusiform gyrus |
| 131 | 47.4 | 41.6 | 13.9 | -4.62 | 0.09 | -7.74 | R middle frontal gyrus, R inferior frontal gyrus (pars triangularis), R inferior frontal gyrus (pars orbitalis), R middle orbital gyrus |
| 112 | -47.3 | 38.3 | 16.1 | -4.68 | 0.10 | -9.09 | L inferior frontal gyrus (pars triangularis), L middle frontal gyrus, L middle orbital gyrus, L inferior frontal gyrus (pars orbitalis) |
| 88 | 5.5 | 59.3 | 21.6 | 4.18 | 0.07 | 6.12 | R superior medial gyrus, L superior medial gyrus, R superior frontal gyrus, R mid orbital gyrus |
| 72 | -26.4 | -85.8 | -52.4 | 4.28 | 0.08 | 6.48 | L cerebellum (crus 2), L cerebellum (crus 1) |
| 71 | -53.5 | -67.9 | -17.3 | -4.49 | 0.10 | -7.14 | L inferior temporal gyrus, L inferior occipital gyrus, L middle temporal gyrus, L middle occipital gyrus |
| 58 | -1.8 | 53.3 | -16.0 | 3.98 | 0.08 | 5.92 | L rectal gyrus, R rectal gyrus, L mid orbital gyrus, L superior orbital gyrus |
| 45 | 55.7 | 8.1 | 25.5 | -4.51 | 0.12 | -7.24 | R inferior frontal gyrus (pars opercularis), R inferior frontal gyrus (pars triangularis) |
| 43 | -7.5 | 58.1 | 23.9 | 4.17 | 0.07 | 5.53 | L superior medial gyrus, L mid orbital gyrus, L superior frontal gyrus |
| 42 | -54.7 | 4.3 | 18.9 | -3.99 | 0.08 | -5.61 | L inferior frontal gyrus (pars opercularis), L inferior frontal gyrus (pars triangularis), L precentral gyrus |
| 36 | 23.8 | -86.5 | -54.0 | 4.27 | 0.14 | 7.24 | R cerebellum (crus 2), R cerebellum (crus 1) |
| 30 | -43.4 | -3.9 | 1.6 | -4.18 | 0.13 | -5.79 | L insula lobe, L superior temporal gyrus, L rolandic operculum, L temporal pole |
| 28 | -30.2 | 38.2 | -17.2 | -4.03 | 0.11 | -5.39 | L inferior frontal gyrus (pars orbitalis), L middle orbital gyrus |
| 23 | -54.0 | 28.7 | -7.6 | 3.86 | 0.06 | 4.59 | L inferior frontal gyrus (pars orbitalis) |
| 23 | 41.8 | -1.9 | 4.5 | -4.04 | 0.12 | -5.39 | R insula lobe, R rolandic operculum, R inferior frontal gyrus (pars opercularis) |
| 22 | 56.8 | 28.3 | -1.0 | 4.36 | 0.13 | 5.64 | R inferior frontal gyrus (pars orbitalis), R inferior frontal gyrus (pars triangularis) |
| 21 | 26.4 | 38.8 | -17.1 | -3.90 | 0.09 | -4.81 | R middle orbital gyrus, R superior orbital gyrus, R inferior frontal gyrus (pars orbitalis) |
| 18 | 45.1 | -43.9 | -28.5 | 4.39 | 0.18 | 5.97 | R fusiform gyrus, R inferior temporal gyrus, R cerebellum (crus 1) |
| 18 | -0.1 | -3.5 | 32.2 | -4.05 | 0.15 | -5.57 | L middle cingulate cortex, R middle cingulate cortex, L anterior cingulate cortex, R anterior cingulate cortex |
| 17 | 41.8 | 2.6 | 47.4 | 3.75 | 0.08 | 4.59 | R middle frontal gyrus |
| 15 | -36.0 | -64.5 | 51.6 | -3.93 | 0.12 | -5.26 | L superior parietal lobule, L inferior parietal lobule |
| 14 | -59.5 | 18.1 | 15.2 | 3.75 | 0.06 | 4.21 | L inferior frontal gyrus (pars triangularis) |
| 13 | 6.5 | -55.8 | -56.6 | 4.63 | 0.27 | 6.10 | R cerebellum (iX), R cerebellum (viii) |

Note: L, left; R, right.

###

### TABLE S3: Univariate TMS effects

| Significant clusters: univariate TMS effects | | | | | | | |
| --- | --- | --- | --- | --- | --- | --- | --- |
| # voxels in cluster | center of mass x | center of mass y | center of mass z | Mean T | SEM T | Max T | Eickhoff-Zilles atlas locations |
| Attention: invalid | | | | | | | |
| Left > Sham | | | | | | | |
| 403 | -3.2 | -67.1 | 24.2 | 2.27 | 0.02 | 3.97 | L precuneus, L cuneus, R precuneus, L middle cingulate cortex, L posterior cingulate cortex, R cuneus, L superior occipital gyrus, L calcarine gyrus, R superior occipital gyrus, R middle cingulate cortex, R posterior cingulate cortex, R calcarine gyrus, L superior parietal lobule |
| Right > Sham | | | | | | | |
| 285 | 2.3 | -63.5 | 23.9 | 2.37 | 0.03 | 4.77 | R precuneus, L precuneus, L posterior cingulate cortex, R middle cingulate cortex, R cuneus, R posterior cingulate cortex, L cuneus, L calcarine gyrus, R calcarine gyrus, L middle cingulate cortex, R superior occipital gyrus |
| Semantics: word | | | | | | | |
| Left > Sham | | | | | | | |
| 202 | 51.8 | -30.3 | 40.6 | -2.17 | 0.03 | -4.12 | R postcentral gyrus, R supramarginal gyrus, R superior temporal gyrus, R precentral gyrus, R inferior parietal lobule, R rolandic operculum, R superior parietal lobule |
| Social cognition: communicative intention | | | | | | | |
| Left > Sham | | | | | | | |
| 226 | -8.5 | -75.7 | -37.9 | -2.31 | 0.04 | -5.27 | L cerebellum (crus 1), cerebellar vermis (6), L lingual gyrus, cerebellar vermis (8), L cerebellum (viii), cerebellar vermis (7), L cerebellum (vii), R lingual gyrus, cerebellar vermis (10), L cerebellum (crus 2), R cerebellum (viii), R cerebellum (vi), cerebellar vermis (9), L cerebellum (iv-v), R cerebellum (crus 2), R cerebellum (iX), cerebellar vermis (4/5) |
| Right > Sham | | | | | | | |
| 1014 | -5.8 | -74.7 | -39.6 | -2.32 | 0.02 | -5.15 | L cerebellum (crus 1), L cerebellum (crus 2), R cerebellum (crus 1), R cerebellum (crus 2), R lingual gyrus, L lingual gyrus, L cerebellum (vi), R cerebellum (vi), L cerebellum (viii), cerebellar vermis (6), L calcarine gyrus, cerebellar vermis (7), R calcarine gyrus, cerebellar vermis (8), R cerebellum (viii), R cerebellum (iX), L fusiform gyrus, R cerebellum (vii), L cerebellum (vii), L inferior temporal gyrus, R fusiform gyrus, R inferior occipital gyrus, L inferior occipital gyrus, cerebellar vermis (4/5), R precuneus, L cerebellum (iX), cerebellar vermis (9), L precuneus, L middle occipital gyrus, R cerebellum (iv-v) |
| 264 | 0.01 | 46.9 | 15.1 | -2.28 | 0.03 | -4.19 | L superior medial gyrus, R anterior cingulate cortex, R superior medial gyrus, L mid orbital gyrus, L anterior cingulate cortex, R mid orbital gyrus, R rectal gyrus, L rectal gyrus, L superior orbital gyrus, L superior frontal gyrus, R middle cingulate cortex, R superior frontal gyrus, R superior orbital gyrus |

Note: L, left; R, right

### TABLE S4. Resting-state IPL connectivity TMS effects

| Significant clusters: resting-state connectivity TMS effects | | | | | | | |
| --- | --- | --- | --- | --- | --- | --- | --- |
| # voxels in cluster | center of mass x | center of mass y | center of mass z | Mean T | SEM T | Max T | Eickhoff-Zilles atlas locations |
| Left IPL (left > sham) | | | | | | | |
| 2027 | 7.5 | -74.0 | -14.0 | -2.28 | 0.01 | -5.48 | R middle occipital gyrus, R superior parietal lobule, R cerebellum (crus 1), L cerebellum (crus 1), L cerebellum (vi), R cerebellum (vi), L middle occipital gyrus, R inferior occipital gyrus, R inferior temporal gyrus, R cerebellum (crus 2), L fusiform gyrus, L inferior occipital gyrus, L cerebellum (crus 2), R precuneus, cerebellar vermis (6), R superior occipital gyrus, R fusiform gyrus, R inferior parietal lobule, R middle temporal gyrus, R cuneus, cerebellar vermis (7), L inferior temporal gyrus, R postcentral gyrus, R lingual gyrus, cerebellar vermis (4/5), R cerebellum (iv-v), L cerebellum (iv-v), R supramarginal gyrus, L middle temporal gyrus, R cerebellum (viii), R cerebellum (vii), cerebellar vermis (8), L cerebellum (viii), L lingual gyrus, R angular gyrus, L cerebellum (vii), R calcarine gyrus, L cerebellum (iii), L cerebellum (iX), R cerebellum (iii) |
| 682 | -14.4 | -61.4 | 40.9 | -2.20 | 0.02 | -3.97 | L superior parietal lobule, L cuneus, L inferior parietal lobule, L precuneus, L superior occipital gyrus, R superior frontal gyrus, L middle cingulate cortex, R SMA, R precuneus, R middle frontal gyrus, L calcarine gyrus, L middle occipital gyrus, R middle cingulate cortex, L paracentral lobule, L postcentral gyrus, L SMA, R cuneus, R paracentral lobule, R precentral gyrus, R calcarine gyrus, L supramarginal gyrus, L angular gyrus |
| 232 | -40.9 | -11.7 | 50.4 | -2.18 | 0.03 | -4.78 | L middle frontal gyrus, L precentral gyrus, L superior frontal gyrus, L postcentral gyrus, L supramarginal gyrus, L SMA, L superior temporal gyrus, L rolandic operculum |
| Right IPL (right > sham) | | | | | | | |
| 242 | 53.8 | -40.2 | 38.5 | -2.19 | 0.03 | -3.81 | R supramarginal gyrus, R postcentral gyrus, R inferior parietal lobule, R superior parietal lobule, R angular gyrus |


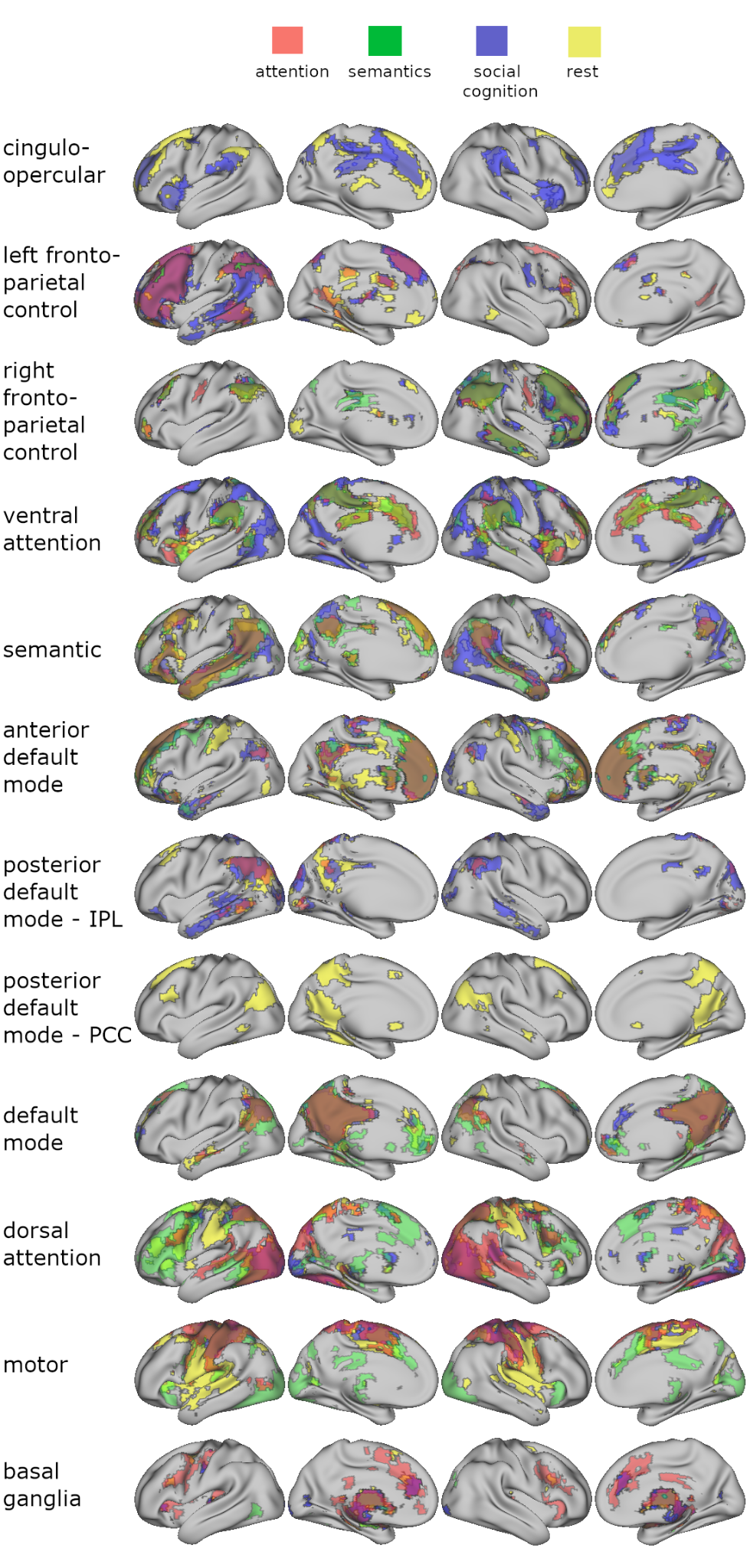


FIGURE S2: Networks organize differently across domains. Masks of large-scale networks derived from each domain are plotted for comparison of spatial topographies across domains. Red: attention, green: semantics, blue: social cognition, yellow: resting state. Masks are generated from one-sided t-tests of ICA components, voxelwise p < 0.001, clusterwise p < 0.05 FWE.


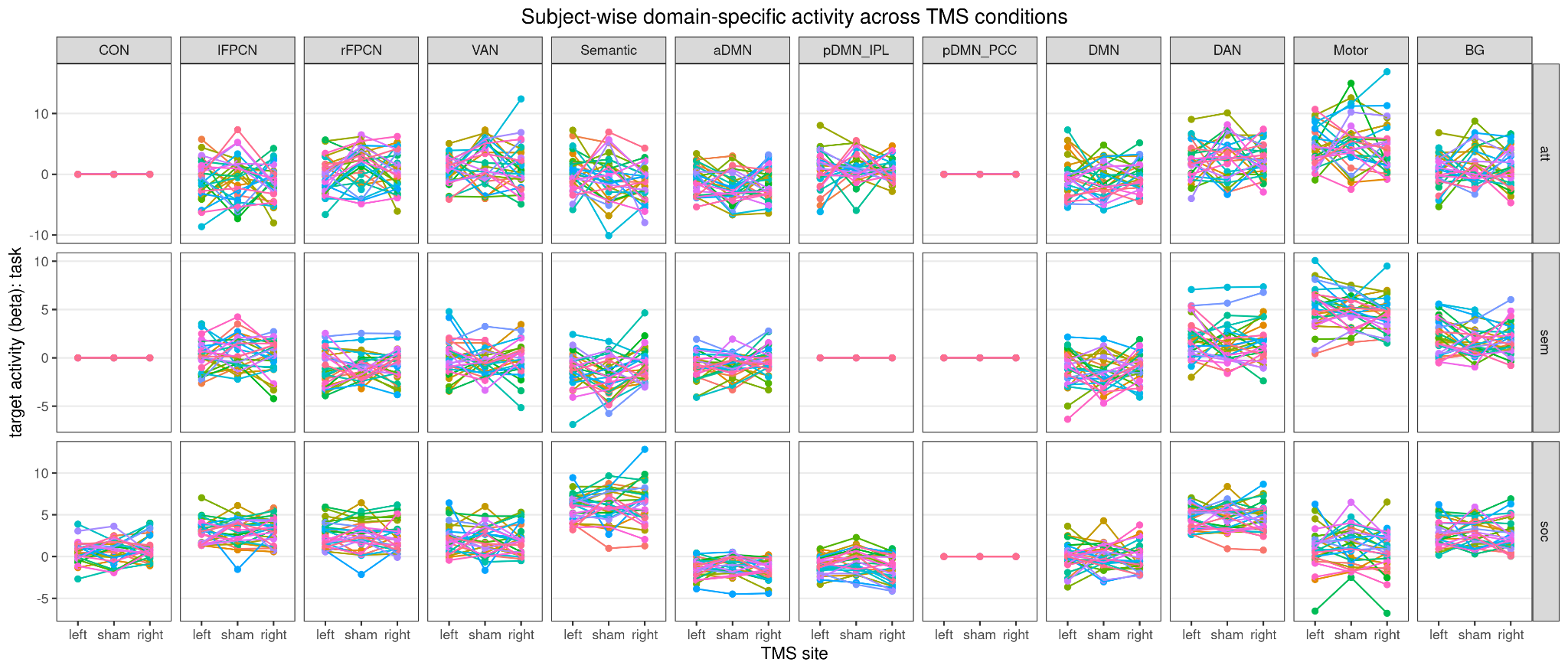


FIGURE S3: Task-specific network activity across TMS conditions. Subject target betas for network activity during the tasks, estimated with multiple temporal regression of the ICA time series in GIFT, are plotted for all resolved networks and TMS conditions

### TABLE S5: Task-specific network activity and TMS effects

|  | CON | lFPCN | rFPCN | VAN | Semantic | aDMN | pDMN-IPL | DMN | DAN | Motor | BG |
| --- | --- | --- | --- | --- | --- | --- | --- | --- | --- | --- | --- |
|  |  |  |  |  |  |  |  |  |  |  |  |
| sham attention (invalid>valid) |  | 0.424 | 1.628 | 2.193 | 0.048 | -0.707 | -0.671 | 0.008 | 2.159 | 0.681 | 0.554 |
| p value |  | 0.486 | **0.009** | **0.001** | 0.945 | 0.188 | 0.145 | 0.989 | **0.000** | 0.136 | 0.333 |
| p corrected |  | 0.607 | **0.031** | **0.007** | 0.989 | 0.313 | 0.291 | 0.989 | **0.003** | 0.291 | 0.476 |
| left attention (invalid L> invalid S) |  | -0.380 | -1.646 | -1.026 | 0.881 | 1.356 | -0.480 | 0.587 | -0.693 | -0.647 | -0.685 |
| p value |  | 0.648 | **0.028** | 0.181 | 0.361 | **0.025** | 0.546 | 0.432 | 0.435 | 0.478 | 0.381 |
| p corrected |  | 0.648 | 0.138 | 0.598 | 0.598 | 0.138 | 0.607 | 0.598 | 0.598 | 0.598 | 0.598 |
| right attention (invalid R> invalid S) |  | -0.830 | -1.158 | -0.684 | -0.976 | 0.713 | -0.384 | 1.045 | -0.468 | -0.920 | -0.255 |
| p value |  | 0.375 | 0.155 | 0.421 | 0.243 | 0.295 | 0.546 | 0.079 | 0.550 | 0.247 | 0.736 |
| p corrected |  | 0.601 | 0.590 | 0.601 | 0.590 | 0.590 | 0.611 | 0.590 | 0.611 | 0.590 | 0.736 |
| sham semantics (word>pseudoword) |  | -0.340 | -0.015 | 0.818 | 0.651 | 0.479 |  | 1.074 | -1.217 | -0.372 | 0.262 |
| p value |  | 0.105 | 0.953 | **0.001** | **0.026** | **0.022** |  | **0.000** | **0.000** | **0.048** | 0.259 |
| p corrected |  | 0.135 | 0.953 | **0.002** | **0.047** | **0.047** |  | **0.000** | **0.000** | 0.073 | 0.291 |
| left semantics (word L> word S) |  | -0.398 | -0.077 | 0.448 | 0.746 | -0.111 |  | 0.421 | 0.435 | 0.338 | 0.626 |
| p value |  | 0.313 | 0.828 | 0.304 | 0.084 | 0.686 |  | 0.338 | 0.301 | 0.326 | 0.077 |
| p corrected |  | 0.434 | 0.828 | 0.434 | 0.376 | 0.772 |  | 0.434 | 0.434 | 0.434 | 0.376 |
| right semantics (word R> word S) |  | -0.438 | 0.276 | 0.393 | 1.295 | 0.606 |  | 0.543 | 0.281 | -0.208 | 0.399 |
| p value |  | 0.212 | 0.406 | 0.276 | **0.003** | 0.068 |  | 0.174 | 0.337 | 0.483 | 0.267 |
| p corrected |  | 0.414 | 0.456 | 0.414 | **0.028** | 0.308 |  | 0.414 | 0.433 | 0.483 | 0.414 |
| sham social cognition (comm. intent > phys. causality) | -1.395 | -0.361 | -1.119 | -2.097 | 4.940 | 2.408 | 1.075 | 1.543 | -1.588 | -0.850 | 0.031 |
| p value | **0.000** | 0.207 | **0.000** | **0.000** | **0.000** | **0.000** | **0.000** | **0.000** | **0.000** | **0.002** | 0.905 |
| p corrected | **0.000** | 0.227 | **0.000** | **0.000** | **0.000** | **0.000** | **0.000** | **0.000** | **0.000** | **0.002** | 0.905 |
| left social cognition (comm. intent L>S) | -0.144 | 0.072 | 0.037 | 0.117 | -0.076 | -0.366 | -0.457 | -0.133 | -0.038 | -0.253 | -0.094 |
| p value | 0.542 | 0.841 | 0.886 | 0.808 | 0.858 | 0.072 | **0.040** | 0.732 | 0.900 | 0.600 | 0.800 |
| p corrected | 0.900 | 0.900 | 0.900 | 0.900 | 0.900 | 0.397 | 0.397 | 0.900 | 0.900 | 0.900 | 0.900 |
| right social cognition (comm. intent R>S) | 0.372 | 0.150 | -0.049 | 0.057 | 0.402 | -0.491 | -0.822 | -0.247 | -0.036 | -0.544 | -0.275 |
| p value | 0.280 | 0.639 | 0.887 | 0.883 | 0.303 | **0.031** | **0.001** | 0.458 | 0.909 | 0.205 | 0.423 |
| p corrected | 0.667 | 0.878 | 0.909 | 0.909 | 0.667 | 0.169 | **0.008** | 0.720 | 0.909 | 0.667 | 0.720 |

Note: Bold values indicate p < 0.05 (10,000 pairwise permutations). Gray areas indicate networks that did not resolve for the domain. For abbreviations, refer to Table S4 note.


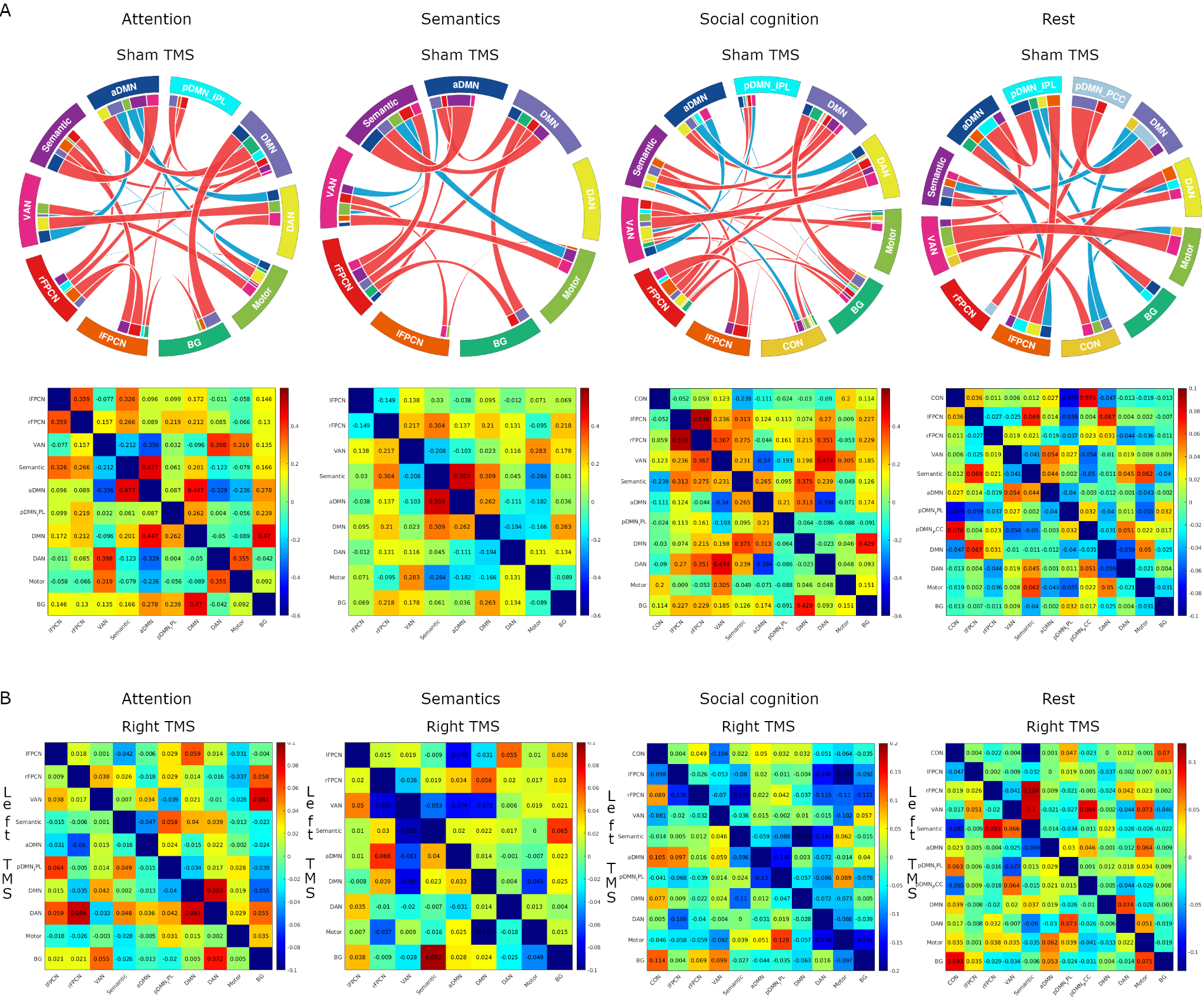


FIGURE S4: Baseline network interactions and TMS effects across domains. (A) Circle plots and matrices in the second row show group mean network interaction values in the sham condition for attention, semantics, and social cognition, and pre-stimulation resting state. Circle plots are thresholded at p < 0.05. (B) Matrices show network connectivity differences between active and sham TMS across domains. The lower triangles of the matrices show left TMS effects and the upper triangles show right TMS effects. Group mean functional connectivity values or differences are displayed for each network pair and condition. Resting state results show active-sham differences of mean pre-post TMS difference matrices.

###

### TABLE S6: TMS effects on large-scale network connectivity

|  | Attention |  |  |  | Semantics |  |  |  | Social cognition |  |  |  | Rest |  |  |  |
| --- | --- | --- | --- | --- | --- | --- | --- | --- | --- | --- | --- | --- | --- | --- | --- | --- |
| Network pair | Left | p value | Right | p value | Left | p value | Right | p value | Left | p value | Right | p value | Left | p value | Right | p value |
| CON-lFPCN |  |  |  |  |  |  |  |  | -0.109 | 0.226 | 0.017 | 0.844 | -0.054 | 0.289 | 0.003 | 0.953 |
| CON-rFPCN |  |  |  |  |  |  |  |  | 0.060 | 0.510 | 0.047 | 0.593 | 0.022 | 0.654 | -0.022 | 0.588 |
| CON-VAN |  |  |  |  |  |  |  |  | -0.072 | 0.396 | -0.105 | 0.248 | -0.020 | 0.647 | -0.008 | 0.863 |
| CON-Semantic |  |  |  |  |  |  |  |  | -0.013 | 0.909 | 0.017 | 0.839 | -0.085 | 0.128 | -0.116 | 0.083 |
| CON-aDMN |  |  |  |  |  |  |  |  | 0.103 | 0.165 | 0.032 | 0.707 | 0.025 | 0.665 | -0.001 | 0.988 |
| CON-pDMN-IPL |  |  |  |  |  |  |  |  | -0.080 | 0.303 | 0.024 | 0.770 | 0.069 | 0.149 | 0.048 | 0.385 |
| CON-pDMN-PCC |  |  |  |  |  |  |  |  |  |  |  |  | -0.072 | 0.117 | -0.027 | 0.539 |
| CON-DMN |  |  |  |  |  |  |  |  | 0.079 | 0.427 | 0.064 | 0.452 | 0.043 | 0.477 | 0.003 | 0.965 |
| CON-DAN |  |  |  |  |  |  |  |  | 0.056 | 0.561 | -0.030 | 0.754 | 0.021 | 0.694 | 0.011 | 0.788 |
| CON-Motor |  |  |  |  |  |  |  |  | -0.040 | 0.685 | -0.061 | 0.451 | 0.039 | 0.344 | 0.005 | 0.896 |
| CON-BG |  |  |  |  |  |  |  |  | 0.111 | 0.161 | -0.012 | 0.900 | 0.096 | 0.081 | 0.071 | 0.126 |
| lFPCN-rFPCN | 0.016 | 0.736 | 0.036 | 0.451 | 0.021 | 0.547 | 0.005 | 0.894 | -0.169 | **0.049** | -0.022 | 0.767 | 0.024 | 0.584 | 0.000 | 0.995 |
| lFPCN-VAN | 0.050 | 0.192 | 0.002 | 0.964 | 0.038 | 0.336 | 0.008 | 0.861 | -0.004 | 0.966 | -0.084 | 0.430 | 0.056 | 0.196 | -0.004 | 0.923 |
| lFPCN-Semantic | 0.006 | 0.911 | -0.030 | 0.595 | 0.031 | 0.438 | -0.014 | 0.725 | -0.018 | 0.818 | -0.140 | 0.111 | -0.016 | 0.786 | -0.032 | 0.492 |
| lFPCN-aDMN | -0.001 | 0.991 | 0.014 | 0.759 | 0.025 | 0.381 | -0.074 | **0.015** | 0.065 | 0.401 | 0.015 | 0.834 | 0.009 | 0.858 | 0.004 | 0.946 |
| lFPCN-pDMN-IPL | 0.038 | 0.326 | 0.013 | 0.770 |  |  |  |  | -0.114 | 0.160 | -0.033 | 0.735 | 0.004 | 0.942 | 0.022 | 0.617 |
| lFPCN-pDMN-PCC |  |  |  |  |  |  |  |  |  |  |  |  | 0.006 | 0.903 | 0.002 | 0.971 |
| lFPCN-DMN | 0.003 | 0.952 | 0.051 | 0.257 | -0.008 | 0.842 | -0.037 | 0.478 | 0.004 | 0.966 | -0.014 | 0.876 | -0.007 | 0.886 | -0.038 | 0.475 |
| lFPCN-DAN | 0.089 | 0.056 | 0.020 | 0.695 | 0.041 | 0.406 | 0.049 | 0.350 | -0.159 | 0.070 | -0.182 | 0.112 | -0.009 | 0.818 | -0.002 | 0.963 |
| lFPCN-Motor | 0.004 | 0.923 | -0.023 | 0.637 | -0.021 | 0.646 | -0.008 | 0.834 | -0.062 | 0.443 | -0.211 | **0.018** | 0.000 | 0.996 | 0.005 | 0.889 |
| lFPCN-BG | 0.007 | 0.858 | -0.021 | 0.579 | 0.028 | 0.533 | 0.022 | 0.662 | 0.003 | 0.974 | -0.105 | 0.174 | 0.034 | 0.415 | 0.012 | 0.697 |
| rFPCN-VAN | 0.006 | 0.881 | 0.035 | 0.479 | -0.071 | 0.090 | -0.038 | 0.431 | -0.038 | 0.654 | -0.097 | 0.354 | -0.015 | 0.742 | -0.042 | 0.214 |
| rFPCN-Semantic | -0.019 | 0.702 | 0.028 | 0.546 | 0.057 | 0.077 | 0.033 | 0.326 | 0.019 | 0.826 | -0.221 | **0.047** | 0.095 | **0.030** | 0.110 | **0.026** |
| rFPCN-aDMN | -0.067 | 0.156 | -0.022 | 0.600 | 0.071 | 0.096 | 0.015 | 0.703 | 0.008 | 0.922 | 0.025 | 0.735 | -0.002 | 0.966 | 0.010 | 0.847 |
| rFPCN-pDMN-IPL | -0.018 | 0.642 | 0.025 | 0.612 |  |  |  |  | -0.075 | 0.349 | 0.001 | 0.994 | -0.017 | 0.638 | -0.021 | 0.611 |
| rFPCN-pDMN-PCC |  |  |  |  |  |  |  |  |  |  |  |  | -0.018 | 0.675 | 0.006 | 0.894 |
| rFPCN-DMN | -0.051 | 0.202 | -0.010 | 0.843 | 0.030 | 0.542 | 0.042 | 0.298 | -0.017 | 0.846 | -0.033 | 0.679 | -0.020 | 0.533 | -0.025 | 0.605 |
| rFPCN-DAN | 0.081 | 0.084 | -0.032 | 0.476 | 0.000 | 0.995 | 0.028 | 0.496 | -0.025 | 0.789 | -0.156 | 0.110 | 0.037 | 0.416 | 0.047 | 0.311 |
| rFPCN-Motor | -0.038 | 0.407 | -0.053 | 0.265 | -0.039 | 0.292 | 0.040 | 0.272 | -0.075 | 0.449 | -0.104 | 0.128 | 0.038 | 0.404 | 0.024 | 0.582 |
| rFPCN-BG | 0.012 | 0.729 | 0.044 | 0.348 | -0.005 | 0.840 | 0.039 | 0.465 | 0.147 | 0.136 | -0.080 | 0.291 | -0.031 | 0.378 | 0.002 | 0.957 |
| VAN-Semantic | 0.006 | 0.898 | 0.005 | 0.901 | -0.078 | **0.028** | -0.049 | 0.287 | 0.032 | 0.695 | -0.075 | 0.467 | 0.070 | 0.096 | 0.108 | **0.011** |
| VAN-aDMN | 0.036 | 0.445 | 0.029 | 0.557 | -0.064 | 0.053 | -0.097 | **0.005** | 0.074 | 0.501 | 0.054 | 0.522 | -0.024 | 0.605 | -0.021 | 0.623 |
| VAN-pDMN-IPL | 0.013 | 0.694 | -0.050 | 0.235 |  |  |  |  | -0.025 | 0.804 | -0.057 | 0.616 | -0.076 | 0.137 | -0.031 | 0.451 |
| VAN-pDMN-PCC |  |  |  |  |  |  |  |  |  |  |  |  | 0.068 | 0.175 | 0.096 | **0.025** |
| VAN-DMN | 0.076 | 0.160 | 0.014 | 0.752 | -0.061 | 0.131 | -0.075 | 0.142 | 0.016 | 0.838 | -0.019 | 0.863 | 0.021 | 0.632 | -0.003 | 0.936 |
| VAN-DAN | -0.040 | 0.353 | -0.017 | 0.619 | -0.015 | 0.735 | 0.010 | 0.854 | 0.044 | 0.686 | -0.009 | 0.916 | -0.010 | 0.848 | -0.046 | 0.416 |
| VAN-Motor | 0.014 | 0.783 | -0.030 | 0.501 | 0.028 | 0.460 | 0.038 | 0.367 | -0.136 | 0.155 | -0.110 | 0.348 | 0.044 | 0.416 | 0.092 | 0.084 |
| VAN-BG | 0.080 | 0.061 | 0.077 | 0.051 | -0.020 | 0.610 | 0.024 | 0.584 | 0.112 | 0.198 | 0.010 | 0.910 | -0.038 | 0.420 | -0.050 | 0.228 |
| Semantic-aDMN | -0.007 | 0.862 | -0.022 | 0.478 | 0.033 | 0.506 | 0.000 | 0.994 | -0.119 | 0.153 | -0.081 | 0.486 | -0.085 | 0.102 | -0.017 | 0.692 |
| Semantic-pDMN-IPL | 0.021 | 0.661 | 0.044 | 0.433 |  |  |  |  | -0.026 | 0.766 | -0.117 | 0.222 | 0.017 | 0.773 | -0.033 | 0.470 |
| Semantic-pDMN-PCC |  |  |  |  |  |  |  |  |  |  |  |  | -0.013 | 0.716 | -0.010 | 0.814 |
| Semantic-DMN | -0.023 | 0.628 | 0.034 | 0.417 | 0.031 | 0.454 | 0.020 | 0.576 | -0.133 | 0.073 | -0.266 | **0.005** | 0.044 | 0.257 | 0.023 | 0.638 |
| Semantic-DAN | 0.068 | 0.060 | 0.039 | 0.347 | 0.002 | 0.962 | 0.027 | 0.480 | -0.022 | 0.812 | -0.176 | 0.059 | -0.066 | 0.212 | -0.033 | 0.509 |
| Semantic-Motor | -0.013 | 0.779 | -0.008 | 0.874 | 0.000 | 0.993 | 0.003 | 0.947 | 0.023 | 0.809 | 0.062 | 0.479 | -0.037 | 0.202 | -0.027 | 0.535 |
| Semantic-BG | -0.055 | 0.211 | -0.040 | 0.278 | 0.091 | **0.049** | 0.062 | 0.205 | -0.002 | 0.981 | -0.013 | 0.870 | -0.007 | 0.844 | -0.024 | 0.560 |
| aDMN-pDMN-IPL | -0.038 | 0.416 | 0.024 | 0.566 |  |  |  |  | -0.150 | 0.108 | -0.169 | **0.030** | 0.046 | 0.183 | 0.039 | 0.400 |
| aDMN-pDMN-PCC |  |  |  |  |  |  |  |  |  |  |  |  | 0.027 | 0.479 | 0.047 | 0.315 |
| aDMN-DMN | -0.032 | 0.507 | -0.020 | 0.596 | 0.012 | 0.841 | -0.002 | 0.962 | -0.020 | 0.834 | -0.005 | 0.957 | 0.022 | 0.632 | 0.000 | 0.998 |
| aDMN-DAN | 0.058 | 0.158 | 0.039 | 0.397 | -0.013 | 0.774 | 0.001 | 0.982 | -0.069 | 0.396 | -0.108 | 0.220 | -0.031 | 0.366 | -0.013 | 0.718 |
| aDMN-Motor | 0.000 | 0.996 | -0.006 | 0.898 | 0.017 | 0.557 | -0.015 | 0.711 | 0.049 | 0.644 | -0.014 | 0.859 | 0.064 | 0.221 | 0.065 | 0.197 |
| aDMN-BG | -0.040 | 0.384 | -0.049 | 0.122 | 0.036 | 0.360 | 0.019 | 0.643 | -0.049 | 0.591 | 0.072 | 0.409 | 0.056 | 0.173 | -0.015 | 0.698 |
| pDMN-IPL-pDMN-PCC |  |  |  |  |  |  |  |  |  |  |  |  | 0.018 | 0.731 | 0.004 | 0.938 |
| pDMN-IPL-DMN | -0.020 | 0.575 | -0.033 | 0.286 |  |  |  |  | -0.047 | 0.602 | -0.050 | 0.572 | -0.028 | 0.597 | 0.009 | 0.841 |
| pDMN-IPL-DAN | 0.044 | 0.346 | 0.016 | 0.641 |  |  |  |  | 0.019 | 0.823 | -0.120 | 0.169 | 0.079 | **0.011** | 0.019 | 0.633 |
| pDMN-IPL-Motor | 0.020 | 0.637 | 0.014 | 0.742 |  |  |  |  | 0.094 | 0.212 | 0.064 | 0.385 | 0.041 | 0.405 | 0.036 | 0.433 |
| pDMN-IPL-BG | -0.011 | 0.791 | -0.037 | 0.332 |  |  |  |  | -0.029 | 0.684 | -0.074 | 0.327 | -0.023 | 0.593 | 0.012 | 0.727 |
| pDMN-PCC-DMN |  |  |  |  |  |  |  |  |  |  |  |  | -0.004 | 0.949 | -0.003 | 0.962 |
| pDMN-PCC-DAN |  |  |  |  |  |  |  |  |  |  |  |  | -0.029 | 0.515 | -0.048 | 0.290 |
| pDMN-PCC-Motor |  |  |  |  |  |  |  |  |  |  |  |  | -0.042 | 0.332 | -0.030 | 0.422 |
| pDMN-PCC-BG |  |  |  |  |  |  |  |  |  |  |  |  | -0.033 | 0.479 | 0.008 | 0.893 |
| DMN-DAN | 0.121 | **0.027** | 0.083 | 0.099 | 0.021 | 0.583 | 0.012 | 0.709 | -0.031 | 0.710 | -0.112 | 0.230 | -0.006 | 0.896 | 0.076 | 0.157 |
| DMN-Motor | 0.032 | 0.554 | 0.013 | 0.719 | -0.084 | **0.046** | -0.055 | 0.171 | -0.105 | 0.262 | -0.099 | 0.312 | -0.036 | 0.407 | -0.032 | 0.429 |
| DMN-BG | 0.007 | 0.828 | -0.082 | **0.031** | 0.045 | 0.350 | 0.016 | 0.752 | -0.017 | 0.810 | 0.014 | 0.851 | 0.002 | 0.954 | -0.004 | 0.940 |
| DAN-Motor | 0.017 | 0.685 | 0.038 | 0.346 | -0.007 | 0.861 | 0.023 | 0.615 | -0.194 | 0.059 | -0.075 | 0.413 | 0.024 | 0.652 | 0.054 | 0.268 |
| DAN-BG | 0.076 | 0.068 | 0.052 | 0.293 | -0.041 | 0.304 | 0.002 | 0.949 | 0.044 | 0.579 | -0.043 | 0.581 | -0.015 | 0.747 | -0.018 | 0.720 |
| Motor-BG | 0.004 | 0.945 | 0.035 | 0.491 | -0.031 | 0.407 | 0.012 | 0.699 | -0.153 | 0.112 | -0.228 | **0.032** | 0.082 | 0.124 | -0.017 | 0.724 |

Note: Bold values indicate p < 0.05 (10,000 pairwise permutations). Gray areas indicate networks that did not resolve for the domain.

### References

[1. Numssen, O., Zier, A.-L., Thielscher, A., Hartwigsen, G., Knösche, T.R., and Weise, K. (2021). Efficient high-resolution TMS mapping of the human motor cortex by nonlinear regression. NeuroImage *245*, 118654. https://doi.org/10.1016/j.neuroimage.2021.118654.](https://www.zotero.org/google-docs/?L41pkY)

[2. Kalloch, B., and Numssen, O. (2022). IMporter. doi:10.13140/RG.2.2.17642.18881.](https://www.zotero.org/google-docs/?L41pkY)

[3. Rothwell, J.C., Hallett, M., Berardelli, A., Eisen, A., Rossini, P., and Paulus, W. (1999). Magnetic stimulation: motor evoked potentials. The International Federation of Clinical Neurophysiology. Electroencephalogr. Clin. Neurophysiol. Suppl. *52*, 97–103.](https://www.zotero.org/google-docs/?L41pkY)

[4. Mayka, M.A., Corcos, D.M., Leurgans, S.E., and Vaillancourt, D.E. (2006). Three-dimensional locations and boundaries of motor and premotor cortices as defined by functional brain imaging: A meta-analysis. NeuroImage *31*, 1453–1474. https://doi.org/10.1016/j.neuroimage.2006.02.004.](https://www.zotero.org/google-docs/?L41pkY)

[5. Silvanto, J., and Pascual-Leone, A. (2008). State-Dependency of Transcranial Magnetic Stimulation. Brain Topogr. *21*, 1–10. https://doi.org/10.1007/s10548-008-0067-0.](https://www.zotero.org/google-docs/?L41pkY)

[6. Hartwigsen, G., Bergmann, T.O., Herz, D.M., Angstmann, S., Karabanov, A., Raffin, E., Thielscher, A., and Siebner, H.R. (2015). Chapter 11 - Modeling the effects of noninvasive transcranial brain stimulation at the biophysical, network, and cognitive Level. In Progress in Brain Research Computational Neurostimulation., S. Bestmann, ed. (Elsevier), pp. 261–287. https://doi.org/10.1016/bs.pbr.2015.06.014.](https://www.zotero.org/google-docs/?L41pkY)

[7. Vanderwal, T., Kelly, C., Eilbott, J., Mayes, L.C., and Castellanos, F.X. (2015). Inscapes: A movie paradigm to improve compliance in functional magnetic resonance imaging. NeuroImage *122*, 222–232. https://doi.org/10.1016/j.neuroimage.2015.07.069.](https://www.zotero.org/google-docs/?L41pkY)

[8. Corbin, N., Todd, N., Friston, K.J., and Callaghan, M.F. (2018). Accurate modeling of temporal correlations in rapidly sampled fMRI time series. Hum. Brain Mapp. *39*, 3884–3897. https://doi.org/10.1002/hbm.24218.](https://www.zotero.org/google-docs/?L41pkY)

[9. Opitz, A., Fox, M.D., Craddock, R.C., Colcombe, S., and Milham, M.P. (2016). An integrated framework for targeting functional networks via transcranial magnetic stimulation. NeuroImage *127*, 86–96. https://doi.org/10.1016/j.neuroimage.2015.11.040.](https://www.zotero.org/google-docs/?L41pkY)

[10. McLaren, D.G., Ries, M.L., Xu, G., and Johnson, S.C. (2012). A generalized form of context-dependent psychophysiological interactions (gPPI): A comparison to standard approaches. NeuroImage *61*, 1277–1286. https://doi.org/10.1016/j.neuroimage.2012.03.068.](https://www.zotero.org/google-docs/?L41pkY)

[11. O’Reilly, J.X., Woolrich, M.W., Behrens, T.E.J., Smith, S.M., and Johansen-Berg, H. (2012). Tools of the trade: psychophysiological interactions and functional connectivity. Soc. Cogn. Affect. Neurosci. *7*, 604–609. https://doi.org/10.1093/scan/nss055.](https://www.zotero.org/google-docs/?L41pkY)

[12. Gitelman, D.R., Penny, W.D., Ashburner, J., and Friston, K.J. (2003). Modeling regional and psychophysiologic interactions in fMRI: the importance of hemodynamic deconvolution. NeuroImage *19*, 200–207. https://doi.org/10.1016/S1053-8119(03)00058-2.](https://www.zotero.org/google-docs/?L41pkY)

[13. Himberg, J., and Hyvarinen, A. (2003). Icasso: software for investigating the reliability of ICA estimates by clustering and visualization. In 2003 IEEE XIII Workshop on Neural Networks for Signal Processing (IEEE Cat. No.03TH8718), pp. 259–268. https://doi.org/10.1109/NNSP.2003.1318025.](https://www.zotero.org/google-docs/?L41pkY)

[14. Salman, M.S., Du, Y., Lin, D., Fu, Z., Fedorov, A., Damaraju, E., Sui, J., Chen, J., Mayer, A.R., Posse, S., et al. (2019). Group ICA for identifying biomarkers in schizophrenia: ‘Adaptive’ networks via spatially constrained ICA show more sensitivity to group differences than spatio-temporal regression. NeuroImage Clin. *22*, 101747. https://doi.org/10.1016/j.nicl.2019.101747.](https://www.zotero.org/google-docs/?L41pkY)

[15. Handwerker, D.A., Gonzalez-Castillo, J., D’Esposito, M., and Bandettini, P.A. (2012). The continuing challenge of understanding and modeling hemodynamic variation in fMRI. NeuroImage *62*, 1017–1023. https://doi.org/10.1016/j.neuroimage.2012.02.015.](https://www.zotero.org/google-docs/?L41pkY)

[16. Xu, J., Calhoun, V.D., Worhunsky, P.D., Xiang, H., Li, J., Wall, J.T., Pearlson, G.D., and Potenza, M.N. (2015). Functional Network Overlap as Revealed by fMRI Using sICA and Its Potential Relationships with Functional Heterogeneity, Balanced Excitation and Inhibition, and Sparseness of Neuron Activity. PLOS ONE *10*, e0117029. https://doi.org/10.1371/journal.pone.0117029.](https://www.zotero.org/google-docs/?L41pkY)

[17. Smith, S.M., Fox, P.T., Miller, K.L., Glahn, D.C., Fox, P.M., Mackay, C.E., Filippini, N., Watkins, K.E., Toro, R., Laird, A.R., et al. (2009). Correspondence of the brain’s functional architecture during activation and rest. Proc. Natl. Acad. Sci. *106*, 13040–13045. https://doi.org/10.1073/pnas.0905267106.](https://www.zotero.org/google-docs/?L41pkY)

[18. Jackson, R.L. (2021). The neural correlates of semantic control revisited. Neuroimage *224*, 117444. https://doi.org/10.1016/j.neuroimage.2020.117444.](https://www.zotero.org/google-docs/?L41pkY)

[19. Yeo, B.T.T., Krienen, F.M., Sepulcre, J., Sabuncu, M.R., Lashkari, D., Hollinshead, M., Roffman, J.L., Smoller, J.W., Zöllei, L., Polimeni, J.R., et al. (2011). The organization of the human cerebral cortex estimated by intrinsic functional connectivity. J. Neurophysiol. *106*, 1125–1165. https://doi.org/10.1152/jn.00338.2011.](https://www.zotero.org/google-docs/?L41pkY)

[20. Fornito, A., Harrison, B.J., Zalesky, A., and Simons, J.S. (2012). Competitive and cooperative dynamics of large-scale brain functional networks supporting recollection. Proc. Natl. Acad. Sci. *109*, 12788–12793. https://doi.org/10.1073/pnas.1204185109.](https://www.zotero.org/google-docs/?L41pkY)
